# Identifying driving mechanisms and threshold effects of trade-offs and synergies among ecosystem services: A case study of Henan Province, China

**DOI:** 10.1371/journal.pone.0347200

**Published:** 2026-04-21

**Authors:** Doudou Dong, Chunyi Li, Jun Du, Xu Yang

**Affiliations:** 1 School of Surveying and Land Information Engineering, Henan Polytechnic University, Jiaozuo, China; 2 Institute of Geographical Sciences, Henan Academy of Sciences, Zhengzhou, China; University of Emergency Management, CHINA

## Abstract

Ecosystem services (ESs) exhibit complex interactions with multiple driving factors, including human activities, climate change, land use, soil erosion, and slope. Henan Province faces ecological challenges such as water scarcity, soil erosion, and biodiversity degradation. Using Henan Province as a case study, this research integrates multi-source data and various models to quantify ESs, identify interrelationships among them, investigate the driving factors influencing these relationships, and determine thresholds for key driving factors from 2000 to 2020. The findings indicate that: (1) ESs in Henan Province showed both trade-offs and synergies. (2) Except for 2010, the key factors of ESs trade-off relationships were only slope, land use; the key factors for the synergies and trade-offs in 2000 and 2020, and the synergies in 2010, were all slope, precipitation (pre), land use, and rainfall erosion (R). Slope, pre, land use, and R maintained strong correlations with ESs synergies and trade-offs throughout the three periods. (3) Land use types showed different correlations with the trade-offs and synergies of ESs pairs, while unused land had no significant correlation. R exhibited two discontinuous sensitive intervals or a single threshold over the three years; slope and pre showed specific sensitive intervals only in 2010 and 2020. Within these thresholds or intervals, the direction of correlation between each factor and ESs pairs reversed.

## 1 Introduction

Ecosystem services (ESs) refer to the various direct and indirect benefits that natural ecosystems provide to humans. Now, the decline in ES capacity is being driven by factors including climate warming, accelerated industrialization, and population growth, resulting in a series of challenges such as water scarcity, soil erosion, increased environmental pollution, diminished carbon sink capacity, and degradation of ecological quality [1–3]. Under these circumstances, the world’s sustainable development is under threat, and ESs assessment has become a global priority. Therefore, clarifying the relationships, driving factors, and threshold effects of ESs has become a focus of ecology and environmental science. And assessment of ESs is a prerequisite for research. Now, the methods for quantitative assessment of ESs are divided into two main categories: the equivalent factor methods and the model calculation methods [4–7]. With the development of science and technology, model calculation methods have become a prominent research area, among which the Integrated Valuation of Ecosystem Services and Trade-offs (InVEST) model has the most applications [8–10].

ESs are not isolated in existence but inherently interconnected, with their core interactions taking two forms: trade-offs and synergies [11–12]. Synergies refer to the improvement of one service promoting the enhancement of another, while trade-offs denote the improvement of one service leading to the decline of another. Shen et al. analyzed the interactive characteristics of trade-offs and synergies among ESs, confirming that their interactions are an inherent attribute of these services [13]. Sun et al. found that the trade-offs and synergies between ESs such as water yield and soil conservation exhibit spatio-temporal variations, and these synergies are primarily observed among regulating services, while trade-offs are mostly found between provisioning services and regulating services [14]. Tian et al. elucidated the trade-off characteristics between food production and regulating services, as well as the stable synergies within regulating services [15]. Clarifying the trade-offs and synergies among ESs is of vital importance for reconciling ecological and developmental goals, and also provides a theoretical reference for regionally specific research.

Trade-offs and synergies in ESs driven by multiple factors, including human activities, natural conditions, and policy interventions [16–18]. The relationships between these driving factors and the trade-offs and synergies of ESs are nonlinear. However, existing studies usually employ linear regression, geographical detectors, and geographically weighted regression methods to explore these driving factors. These methods overlook the nonlinear impacts and are primarily qualitative analyses, failing to reveal the complex interaction mechanisms among ESs [19–21]. Ecosystems are highly uncertain, with fluctuating natural elements and complex human activities that correlate with changing trade-offs and synergies in ESs. The Bayesian Belief Network (BBN) depicts the nonlinear relationships using the conditional probability distribution among nodes, fits the probability functions through observations and experiments, and restores the dynamic impacts of elements on service interactions. It uses Bayes’ theorem to optimize node probabilities based on real-time information and builds a flexible and practical analytical framework for researchers to excel in uncertainty quantification. In addition, it can efficiently integrate multi-source data and provide strong support for ES research [13,22–25]. This study employs BBNs rather than the XGBoost-SHAP (SHapley Additive exPlanations) approach to identify key associated factors in ESs. While the XGBoost-SHAP method can detect statistical associations between variables, it operates as a post-hoc analysis tool and cannot establish causal pathways or conditional dependency structures among variables [26]. In contrast, BBNs directly learn network-based associative relationships from data. The resulting graphical models provide interpretable representations of variable interactions, suggesting potential causal links based on structural associations without asserting definitive causal relationships [27–28]. Instead, its strengths in nonlinear modeling and interpretability are better suited for threshold analysis, which is addressed separately in this study.

Notably, the trade-offs and synergies among ESs do not change continuously but may exhibit critical thresholds: specific value intervals of driving factors within which the interaction dynamics on service relationships shift abruptly, such as transitioning from synergy to trade-off and vice versa [29–30]. The presence of such threshold effects renders traditional linear models (e.g., segmented regression) inadequate for capturing the dynamic transitions in ES relationships, necessitating the introduction of quantitative methods capable of characterizing nonlinear relationships and identifying critical thresholds. For this specific threshold analysis objective, the XGBoost-SHAP model is employed in the present study: it integrates the nonlinear modeling advantages of XGBoost, an advanced and regularized implementation of the Gradient Boosted Decision Trees (GBDT) framework, with the interpretability of SHAP, enabling the effective capture of nonlinear relationships between driving factors and ES trade-offs and synergies. By calculating SHAP values for feature variables, this model quantifies the contribution of each factor to relationship transitions and pinpoints threshold intervals with precision [31–33]. Compared to these traditional linear alternatives and basic GBDT, it not only handles high-dimensional data and complex interactions but also visually presents the marginal effects of associated factors and threshold locations through visualization tools.

Henan Province has typical regional characteristics, such as strong geomorphological differentiation, diverse functional patterns of land use, which together exert significant pressure on its ecosystems. On the one hand, the diversity of geomorphology makes different regions rich in ecosystem types but with varying degrees of instability. On the other hand, complex land use patterns mean that ESs are intertwined and associated with each other. Ecosystems are inherently fragile and face many challenges due to the double disturbance of natural factors and human activities. Relevant research on Henan Province or its subregions has achieved remarkable progress. Most studies have focused on the spatio-temporal heterogeneity of ESs [34–35]; some have attempted to identify the driving factors of the trade-offs and synergies of ESs via linear analysis or BBN [13–15,36]. However, these studies have limitations: the use of linear methods makes it difficult to characterize the complex nonlinear relationships between driving factors and ESs; even studies on driving mechanisms based on BBN only conduct analyses for a single year and thus fail to reveal the temporal heterogeneity of such mechanisms [13]; meanwhile, most studies have not achieved the accurate quantification of the thresholds of key factors [37–38]. Therefore, an in-depth study of driving factors, and threshold effects of ESs trade-offs and synergies in Henan Province is crucial for policy implementation, ecosystem management optimization, and achieving regional ecological sustainability.

Henan Province, as a major national grain production area and the core area of the Yellow River Basin, is confronted with complex ecological challenges. This study selects seven key ESs for targeted quantification. Carbon storage (CS) aligns with the national “dual-carbon” strategy; the sediment delivery ratio (SDR) is used to characterize the soil erosion characteristics in the western mountainous areas and the sediment accumulation risk in the eastern plain; habitat quality (HQ) responds to the urgent demand for biodiversity conservation; nitrogen (N) and phosphorus (P) export reflects the water quality pressure caused by agricultural activities; water yield (WY) addresses the issue of temporal and spatial heterogeneity of water resources; food supply (FS) provides a solid underpinning for national food security, with Henan being a major agricultural province in China. Taking Henan Province as the study area, this study systematically quantifies the aforementioned seven key ESs. It uses correlation analysis to explore the trade-offs and synergies between ESs and changes from 2000 to 2020. It constructs a Bayesian Belief Network-Ecosystem Services model (BBN-ESs) to explore nonlinear relationships with key factors and further introduces the XGBoost-SHAP model to conduct threshold analysis on dominant driving factors, aiming to identify critical thresholds intervals where service relationships undergo significant shifts. This study examines the period 2000–2020 and aims to (1) discriminate the interrelationships of ESs in Henan Province; (2) identify the key factors driving ESs trade-offs and synergies; (3) determine the thresholds of dominant driving factors.

## 2 Materials and methods

### 2.1 Study area

**Fig 1:**
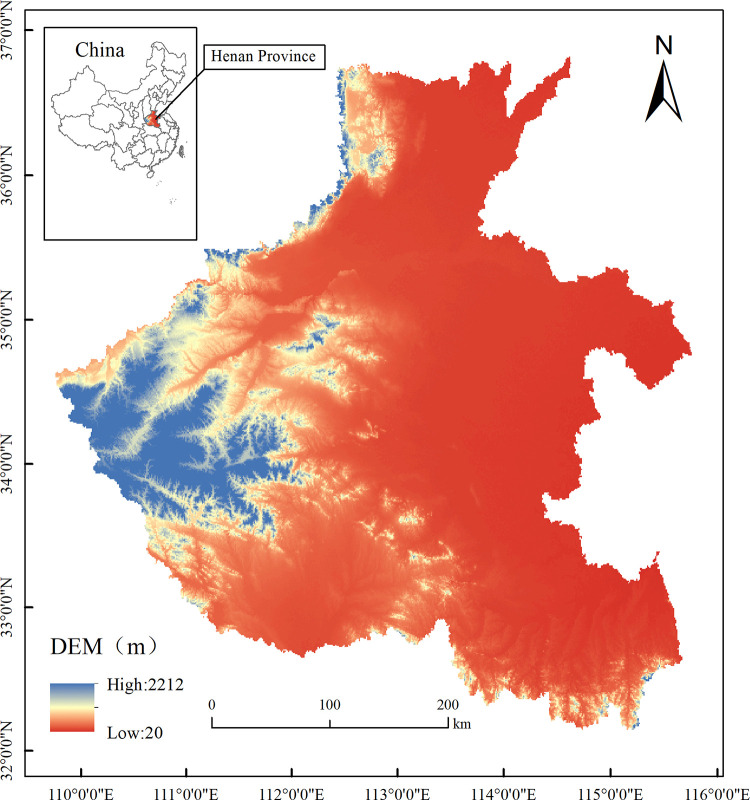
Schematic of the study area.

Henan Province (110°21′–116°39′ E, 31°23–36°22′ N) is located in central China (Fig 1). The terrain exhibits higher elevations in the west and lower elevations in the east. The western region, dominated by the Taihang and Funiu Mountains, consists of hilly and mountainous areas with rich vegetation and high biodiversity, serving as an important water conservation area. In contrast, the eastern plains are vast and flat, representing the primary agricultural and living areas where human activities significantly influence ecosystems. The climate is characterized by a temperate continental monsoon type, with annual precipitation ranging from 500 to 900 mm and an average annual temperature generally between 12℃ and 16℃.

### 2.2 Data sources

The data required for this study primarily include land use, the Digital Elevation Model (DEM), meteorological data, population density data, soil data, and the Normalized Difference Vegetation Index (NDVI) (Table 1). Carbon pool data for calculating carbon storage are from relevant research papers [39–41]. The parameters required for the InVEST model, including habitat suitability coefficients and threat source weights, are detailed in the InVEST Model User’s Manual (Version 3.14.1). Data such as rainfall erosivity, soil erodibility factor, soil depth, and plant available water content were calculated based on meteorological and soil data using the Spatial Analysis, Attribute Extraction, and Raster Calculator tools in ArcGIS 10.8. Slope data was derived from DEM data via the Slope tool in ArcGIS 10.8. To ensure the precise alignment of all datasets at the pixel level, all data were resampled to a spatial resolution of 1 km using the Resample tool in ArcGIS 10.8, and the original projected coordinate system was retained with the Project tool.

**Table 1:** Data sources and specifications.

Data type	Time	Resolution	Source
Vector Data	—	—	Natural Earth (https://www.naturalearthdata.com/)
Land Use	2000, 2010, 2020	30m	Resources and Environmental Sciences Data Platform (http://www.resdc.cn)
DEM	—	90m	The data set is provided by Geospatial Data Cloud site, Computer Network Information Center, Chinese Academy of Sciences. (http://www.gscloud.cn)
Meteorological	2000-2020	1km	National Earth System Science Data Center(http://www.geodata.cn)
Population density	2000, 2010, 2020	1km	World Population Prospects(http://www.worldpop.org)
NDVI	2000, 2010, 2020	1km	Resource and Environmental Science Data Center (http://www.resdc.cn)
Soil data	—	1:1000000	National Cryosphere Desert Data Center (http://www.ncdc.ac.cn/portal/)

### 2.3 Methods

#### 2.3.1 ESs assessment.

In this study, the WY, SDR, CS, N, P, and HQ modules in the InVEST model 3.14.1 were used to evaluate the ESs in the study area. FS were calculated using mathematical statistics methods.

(1) water yield

Water yield was calculated using the “Water Yield” module of the InVEST model 3.14.1. The formula is as follows:


$$Y(x)=(\text{1}-\frac{AET(x)}{P(x)})\times P(x)$$ (1)

Where Y(x), P(x), and AET(x) represent the annual water yield (mm), annual precipitation (mm), and annual actual evapotranspiration (mm) of grid cell *x*.

(2) sediment delivery ratio

Based on the universal soil loss equation (USLE) theory, the soil conservation amount in the regulating service is obtained using the “Sediment Delivery Ratio” module in the InVEST model 3.14.1. The formulas are as follows:


PSLA=R×K×LS (2)

USLE=R×K×C×P (3)

SEDRET=PSLA−USLE (4)

Where PSLA (potential soil loss amount) represents the potential soil loss amount (t/(ha·a)); USLE (actual soil loss amount) represents the actual soil loss amount (t/(ha·a)); *R*, *K*, *LS*, *C*, and *P* represent the rainfall erosion (MJ·mm/(ha·h·a)), soil erodibility (t·ha·h/(ha·MJ·mm)), topographic factor (slope length and steepness, dimensionless), vegetation cover and management factor (dimensionless), and soil conservation practice factor (dimensionless), respectively; and SEDRET (sediment retention) represents the amount of soil conservation (t/(ha·a)).

(3) carbon storage

The carbon storage amount in the regulating service is calculated through the “Carbon Storage and Sequestration” module in the InVEST model 3.14.1. The formula is as follows:


$$C_{total}=C_{above}+C_{below}+{C_{soil}+C}_{dead}$$ (5)

Where $C_{total}$ represents the total carbon stock (t/ha), $C_{above}$ represents the aboveground carbon stock (t/ha), $C_{below}$ represents the belowground carbon stock (t/ha), $C_{soil}$ represents the soil carbon stock (t/ha), and $C_{dead}$ represents the dead organic matter carbon stock (t/ha).

(4) nutrient delivery ratio

Through the “Nutrient Delivery Ratio” module in the InVEST model 3.14.1, and the export amounts of nitrogen (N) and phosphorus (P) are calculated. Under this evaluation framework, lower outputs of nitrogen and phosphorus indicate a stronger water purification capacity. The formula is as follows:


$$X\_{export}_{n}=load(X{)}_{n}\times {NDR(X)}_{n}$$ (6)

Where $X\_{export}_{n}$ represents the N or P export of grid cell *n* ($\text{kg}\cdot {\text{pixel}}^{-\text{1}}$); $load(X{)}_{n}$ represents the modified N or P load of grid cell *n* (kg); ${NDR(X)}_{n}$ represents the N or P delivery rate of grid cell *n* (dimensionless); and *X* represents N or P.

(5) habitat quality

The habitat quality is calculated using the “Habitat Quality” module in the InVEST model 3.14.1. The formula is as follows:


$$Q_{xj}=H_{j}\times (\text{1}-\frac{\overset{z}{\underset{xj}{D}}}{K^{Z}+\overset{Z}{\underset{xj}{D}}})$$ (7)

Where $Q_{xj}$ represents the habitat quality of pixel *x* in land use type *j* (dimensionless); $H_{j}$ represents the habitat suitability of land use type *j* (dimensionless); $\overset{z}{\underset{xj}{D}}$ represents the weighted average of threat sources of pixel *x* in land use type *j* (dimensionless); *z* is a constant with a value of 2.5 (dimensionless); and *K* is a constant with a value of 0.5 (dimensionless).

(6) food supply

Previous studies have shown a significant linear relationship between food supply and NDVI. This relationship was quantified to calculate food supply using the Raster Calculator tool in ArcGIS 10.8 software, with the calculation formula as follows:


$${FS}_{x}=\frac{{NDVI}_{x}}{{NDVI}_{sum}}\times {FS}_{sum}$$ (8)

Where ${FS}_{x}$ represents the grain yield of grid *x* (t), ${FS}_{sum}$ represents the total grain yield of Henan Province in the current year (t), ${NDVI}_{x}$ represents the NDVI of grid *x* (dimensionless), and ${NDVI}_{sum}$ represents the total NDVI of cropland in Henan Province (dimensionless).

#### 2.3.2 ESs trade-offs and synergies.

The trade-offs and synergies among ESs are calculated using the Spearman coefficient. The formula is as follows:


$$\begin{array}{c} r=\frac{\frac{\text{1}}{n}\sum_{i=\text{1}}^{n}\left(R\left(x_{i}\right)-\overset{―}{R(x)}\right)\times \left(R\left(y_{i}\right)-\overset{―}{R(y)}\right)}{\sqrt{\frac{\text{1}}{n}\sum_{i=\text{1}}^{n}{\left(R\left(x_{i}\right)-\overset{―}{R(x)}\right)}^{\text{2}}}\times \left(\frac{\text{1}}{n}\sum_{i=\text{1}}^{n}{\left(R\left(y_{i}\right)-\overset{―}{R(y)}\right)}^{\text{2}}\right)} \end{array}$$ (9)

Where $R\left(x_{i}\right)$ and $R\left(y_{i}\right)$ are the ranks of $x_{i}$ and $y_{i}$, respectively; $\overset{―}{R(x)}$ and $\overset{―}{R(y)}$ are the average ranks of variables *x* and *y,* respectively. *r* is the correlation coefficient between variables *x* and *y*. The value range of *r* is [- 1, 1]. As |r| increases, the correlation becomes stronger. When *r >* 0, it indicates synergy between the variables; when *r* < 0, it indicates a trade-off between the variables. Based on this principle, this study employs the “Spearman” method in the “corrplot” package of RStudio 2024.04.2 + 764 to conduct Spearman correlation analysis.

Given the multiple correlation tests performed across all pairs of ESs, we applied the Benjamini-Hochberg procedure to control the False Discovery Rate (FDR). The significance of correlations reported in the main text, along with the sample sizes and FDR-adjusted p-values, is indicated by asterisks in the figures. Both the raw p-values,95% confidence intervals and the FDR-corrected p-values have been provided in S1-S2 Tables (S1 Appendix) for full transparency.

#### 2.3.3 Analysis of the driving mechanisms for trade-offs and synergies in ESs.

##### 2.3.3.1 Theory of Bayesian belief network

Bayesian belief network (BBN), proposed by Pearl in 1988, is a probabilistic graphical model combining probability and graph theories. It can be used to analyze the uncertain relationships among variables and is also known as a belief network. It is a directed acyclic graph whose directed edges represent the conditional dependencies among the variables, and each node represents a variable. Moreover, each node contains the conditional probability table specifying its probability distribution. The conditional probabilities of nodes reflect the strength of the conditional dependencies between parent nodes and child nodes. The formula is as follows:


$$P\left(Y|X\right)=\frac{P(XY)}{P(X)}$$ (10)

Where P(Y|X) represents the probability of the occurrence of event *Y* under the condition that event *X* has already occurred; P(XY) represents the probability of the simultaneous occurrence of events *X* and *Y*; and P(X) represents the prior probability of event *X*.

**2.3.3.2 Analysis of the importance of driving factors for ESs:** Based on the sensitivity analysis using Netica 5.18 software, the relative importance of the driving factor nodes to the ESs nodes in the constructed model is explored by calculating the variance reduction (VR). This is because VR can quantify the extent to which a specific driving factor reduces the uncertainty (measured by variance) of ESs nodes when the factor’s evidence is updated: the higher the VR value, the stronger the factor's ability to reduce the uncertainty of ESs, thus enabling the identification of key driving factors based on this. The calculation formula is:


$$VR=V(Q)-V\left(Q|F\right)=\sum_{q}p(q)\times {\left[X_{q}-E(Q)\right]}^{\text{2}}-\sum_{q}p\left(q|f\right)\times {\left[X_{q}-E\left(Q|F\right)\right]}^{\text{2}}$$ (11)

Where *VR* represents the variance reduction, indicating the magnitude of relative importance; V(Q) and E(Q) are the variance and expectation of the ES *Q,* respectively; V(Q|F) and E(Q|F) represent the variance and expectation of ES *Q* under the condition of variable *F*, respectively; $X_{q}$ represents the actual value corresponding to state *q*. A higher *VR* value indicates that the node has greater relative importance to the target node, meaning it has a stronger influence on the ES node; conversely, a lower *VR* value suggests a weaker influence on the ES node.

**2.3.3.3 Scenario simulation design for BBN-ESs model:** By setting different state probability values of ESs nodes in the BBN-ESs Model as scenarios, probabilistic inference was used to calculate the relative changes in posterior probabilities compared to prior probabilities for key node states affecting ESs under each scenario. Based on the trade-offs and synergies among ESs, four scenarios were designed:

Scenario I: Maximize ESs A and B with synergies by setting their “High=100%”;

Scenario II: Minimize ESs A and B with synergies by setting their “Low=100%”;

Scenario III: Maximize ES A and minimize ES B with a trade-off by setting ES A to “High=100%” and ES B to “Low=100%”;

Scenario IV: Minimize ES A and maximize ES B with a trade-off by setting ES A to “Low=100%” and ES B to “High=100%”.

Combining the four scenario classifications with the trade-offs and synergies among ESs in 2000, 2010, and 2020, a detailed classification of each scenario is presented in Tables 2–3.

**Table 2:** Classification of Scenario I and Scenario II in 2000, 2010, and 2020.

Scenarios	2000(Scenario I、Scenario II)	2010(Scenario I、Scenario II)	2020(Scenario I、Scenario II)
State		
1	FS、N、P、WY highest = 100%	WY、SDR、HQ、CS highest = 100%	SDR、HQ、CS highest = 100%
2	FS、N、P、WY low = 100%	WY、SDR、HQ、CS low = 100%	SDR、HQ、CS low = 100%
3	CS、FS highest = 100%	CS、FS highest = 100%	N、P、WY、FS highest = 100%
4	CS、FS low = 100%	CS、FS low = 100%	N、P、WY、FS low = 100%
5	SDR、WY、CS highest = 100%	N、P、WY、FS highest = 100%	SDR、WY highest = 100%
6	SDR、WY、CS low = 100%	N、P、WY、FS low = 100%	SDR、WY low = 100%
7	SDR、HQ highest = 100%		
8	SDR、HQ low = 100%		
9	CS、HQ highest = 100%		
10	CS、HQ low = 100%		

**Table 3:** Classification of Scenario III、Scenario IV in 2000, 2010, and 2020.

Scenarios	2000(Scenario III、Scenario IV)	2010(Scenario III、Scenario IV)	2020(Scenario III、Scenario IV)
State		
1	N low = 100% HQ、SDR、CS highest = 100%	N low = 100% HQ、SDR、CS highest = 100%	N low = 100% HQ、SDR、CS highest = 100%
2	N highest = 100% HQ、SDR、CS low = 100%	N highest = 100% HQ、SDR、CS low = 100%	N highest = 100% HQ、SDR、CS low = 100%
3	P low = 100% HQ、SDR、CS highest = 100%	P low = 100% HQ、SDR、CS highest = 100%	P low = 100% HQ、SDR、CS highest = 100%
4	P highest = 100% HQ、SDR、CS low = 100%	P highest = 100% HQ、SDR、CS low = 100%	P highest = 100% HQ、SDR、CS low = 100%
5	WY low = 100% HQ highest = 100%	FS low = 100% HQ、SDR highest = 100%	FS low = 100% HQ、SDR、CS highest = 100%
6	WY highest = 100% HQ low = 100%	FS highest = 100% HQ、SDR low = 100%	FS highest = 100% HQ、SDR、CS low = 100%
7	FS low = 100% HQ、SDR highest = 100%		WY low = 100% HQ、CS highest = 100%
8	FS highest = 100% HQ、SDR low = 100%		WY highest = 100% HQ、CS low = 100%

**2.3.3.4 Statistical Identification of Drivers for ESs Trade-offs and Synergies:** To identify primary associated factors, we computed the absolute changes between posterior and prior probabilities using Python 3.7, with data processing supported by the pandas package and bootstrap resampling implemented via the bootstrap function. Any factor with a change greater than the 95th percentile of these values was deemed a driver. The confidence interval was estimated using the bootstrap method: we generated 10,000 bootstrap samples by resampling with replacement and calculated the 95th percentile for each sample, ultimately deriving the 95% confidence interval (CI) for this threshold.

#### 2.3.4 XGBoost-SHAP.

**2.3.4.1 XGBoost:** The XGBoost model is an algorithm based on gradient-boosted decision trees. It combines multiple weak decision trees into a strong classifier by continuously splitting, pruning, and boosting classification trees. Each tree is trained based on the residuals of the previous tree, gradually optimizing the loss function to reduce errors [42]. Meanwhile, it controls the complexity of the trees and introduces regularization (such as the regularization mechanism that is included by default in the model but not explicitly written in the code) to reduce the risk of overfitting. This model was implemented in Python 3.7, via the xgboost package. During model training, the XGBClassifier class of the xgboost package was adopted, with its *fit* method is used, taking the features of the training set *x_train* and the target variable *y_train* as inputs. Finally, a trained classification model is obtained for subsequent analysis of the test set. To ensure model precision, we performed parameter tuning using grid search coupled with three-fold cross-validation. The macro-average F1-score (F1-macro) was selected as the optimization metric to accurately identify the optimal parameter set. We focused on optimizing five key parameters: n_estimators, max_depth, learning_rate, subsample, and colsample_bytree. After model selection, we employed the Area Under the Curve (AUC), accuracy, F1-score, and the F1-score for each class as evaluation metrics to ensure a comprehensive assessment of predictive performance across all classification tasks.

XGBoost is an additive model, and its prediction results are obtained by the weighted sum of a series of weak classifiers (trees). For sample *i*, the predicted value at the *t-th* iteration is:


$${\hat{y_{i}}}^{\left(t\right)}={\hat{y_{i}}}^{\left(t-\text{1}\right)}+f_{t}\left(x_{i}\right)$$ (12)

Where ${\hat{y_{i}}}^{\left(t-\text{1}\right)}$ is the predicted value at the *(t-1)-th* iteration, and $f_{t}\left(x_{i}\right)$ is the prediction of the *t-th* tree for sample *i*. During training, XGBoost optimizes the model by minimizing the following loss function:


$$\begin{array}{c} L^{\left(t\right)}=\sum \overset{n}{\underset{i=\text{1}}{\mathrm{\backslash nolimits}}}l\left(y_{i},{\hat{y_{i}}}^{\left(t-\text{1}\right)}+f_{t}\left(x_{i}\right)\right)+\Omega \left(f_{t}\right) \end{array}$$ (13)

Where $l\left(y_{i},{\hat{y_{i}}}^{\left(t-\text{1}\right)}+f_{t}\left(x_{i}\right)\right)$ is the loss for sample *i,* $\Omega \left(f_{t}\right)=\gamma T+\frac{\text{1}}{\text{2}}\lambda \sum_{j=\text{1}}^{T}\overset{\text{2}}{\underset{j}{\omega }}$is the regularization term to control tree complexity, *T* is the number of leaf nodes in the tree, $\omega_{j}$ is the weight of leaf node *j*, and γ and λ are regularization parameters.

**2.3.4.2 SHAP:** SHAP (SHapley Additive exPlanations) is a model interpretation method based on the Shapley Value from cooperative game theory, whose core idea is to fairly allocate the contribution of each feature to the model's prediction result, ensuring that the impact of each feature can be reasonably measured. Conceptually, for a machine learning model, SHAP values assign a numerical value to each feature, representing the magnitude and direction of the feature's influence on the model's prediction. SHAP analyses were implemented in Python 3.7 via the shap package. In the binary classification scenario, SHAP values measure the contribution of features to the probability of class 1 (synergy):

The calculation of SHAP values is based on the following formula (taking feature *i* as an example):


$$\Phi_{i}=\sum_{S\subseteq \left\{\text{1},\text{2},....,M\right\}\backslash \left\{i\right\}}\frac{|S|!\left(M-|S|-\text{1}\right)!}{M!}[f_{x}\;\;\;\;\left(S\cup \left\{i\right\}\right)-f_{x}(S)]$$ (14)

Where *M* is the total number of features, *S* is a feature subset excluding feature *i*, and $f_{x}(S)$ is the model's output when only the features in subset *S* are used. This formula calculates the contribution of feature *i* to the prediction (i.e., the SHAP value) by iterating over all possible combinations of feature subsets, computing the difference in model output before and after adding feature *i*, and taking a weighted average of these differences based on the size of the subsets.

**2.3.4.3 Binning and LOESS smoothing method:** To address the issue of unclear trends in SHAP values for continuous features caused by data discreteness, this study adopts a combined approach of the binning method and Locally Weighted Scatterplot Smoothing (LOESS). This approach was implemented in Python 3.7 via the pandas and statsmodels packages. This method extracts the underlying relationship patterns between feature values and SHAP values, providing a stable trend reference for subsequent threshold determination. The binning method first divides the continuous feature values into several intervals based on quantiles, and calculates the mean feature value, mean SHAP value, and confidence interval for each interval, thereby achieving data aggregation and reducing noise from individual samples. On this basis, the LOESS smoothing algorithm fits the binned results through locally weighted regression, generating a continuous curve that eliminates inter-bin fluctuations and clearly reveals the overall trend between features and SHAP values.

### 2.4 Research framework

**Fig 2:**
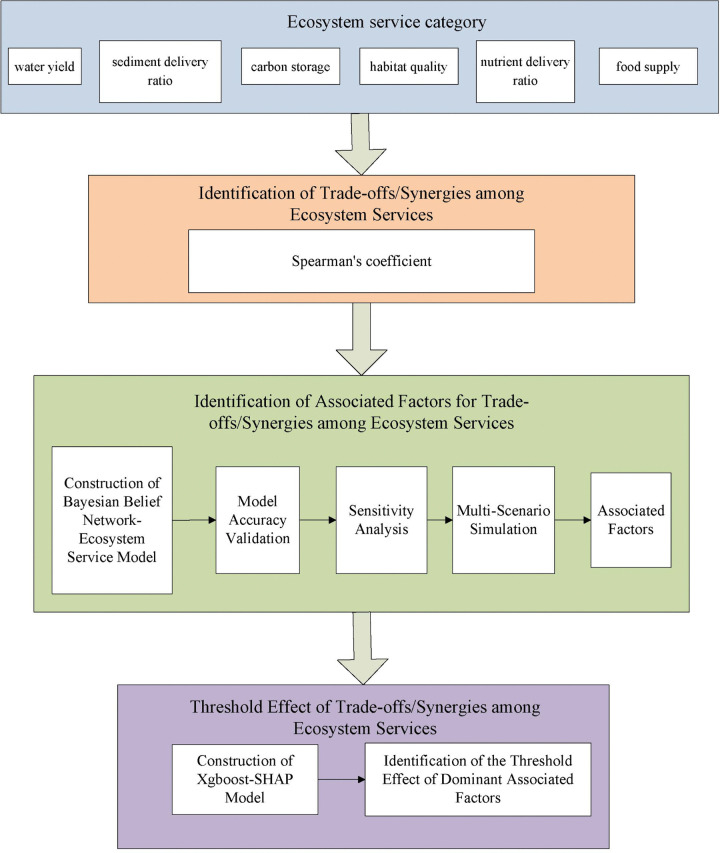
Research framework.

Based on the research content, which includes the identification of trade-offs/synergies among ESs, the identification of driving factors, and the study on threshold effects, the research framework figure of this study is shown below Fig 2.

## 3 Results

### 3.1 Trade-offs and synergies of ESs

**Fig 3:**
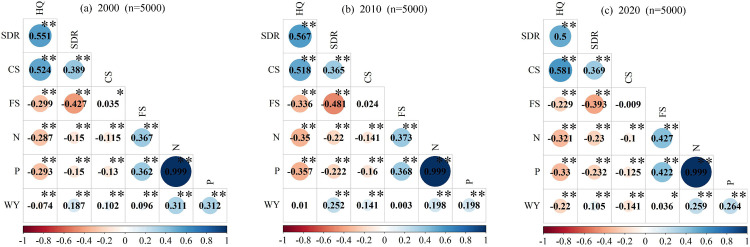
Correlation coefficients (a, b, c) of ESs in Henan Province from 2000 to 2020. Statistical significance was denoted using an asterisk system (*, **) to represent FDR-adjusted significance levels, as detailed in the figure legend (e.g., *p < 0.05; ** p < 0.01).

From 2000 to 2020, the relationships among ESs in Henan Province were characterized by the coexistence of synergies and trade-offs (Fig 3a–3c). Most of these synergies and trade-offs passed the statistical significance test, with only the WY-HQ, WY-FS, and CS-FS pairs in 2010, and the CS-FS pair in 2020 failing to meet the significance level.

The N-P pair consistently showed a strong synergy; HQ-CS and HQ-SDR consistently exhibited moderate synergies; FS-N/P shifted from a weak synergy to a moderate synergy; SDR-CS, WY-SDR, WY-FS, and WY-N/P consistently showed weak synergies; HQ-N/P, HQ-FS, SDR-N/P, and CS-N/P consistently exhibited weak trade-offs; SDR-FS shifted from a moderate trade-off to a weak trade-off. CS-FS shifted from a weak synergy to a weak trade-off, WY-HQ shifted from a weak trade-off to a weak synergy and then back to a weak trade-off, and WY-CS shifted from a weak synergy to a weak trade-off.

### 3.2 Analysis of the driving factors for the trade-offs and synergies of ESs

#### 3.2.1 Construction and accuracy testing of the Bayesian belief network-Ecosystem services Model.

This study used Netica 5.18 software to construct the BBN-ESs Model. Detailed model construction methods are described in S2 Appendix.

This study evaluated the model’s accuracy using an error matrix. The original data were randomly split into a 70% training set and a 30% test set using simple random sampling in Python 3.7 for model accuracy assessment. The accuracy of the seven target nodes was tested using Netica 5.18 software, where the error matrix was calculated to assess model accuracy. The accuracy of the seven target ESs nodes exceeded 60%, with overall accuracy of 76.57%, 76.15%, and 76.07% for the three years, respectively. Detailed accuracy data are presented in S2 Fig, which is included in S3 Appendix. This indicates that the model has high accuracy and high reliability in predicting the probabilities of the seven ES nodes.

#### 3.2.2 Sensitivity analysis and driving factor screening.

By calculating the VR of each factor on each type of ES, we can identify the most influential factors on ESs. The calculated results are plotted, as shown in S3 Fig (included in S3 Appendix). As shown in S3 Fig, from 2000 to 2020, the key nodes influencing N/P included land use, precipitation (pre), rainfall erosion (R), soil erosion (k), NDVI, and population (POP). Land use had the highest VR proportion, indicating the most significant impact on N/P changes. The key nodes influencing HQ included land use, k, NDVI, and POP. Land use had the highest VR proportion, indicating the most significant impact on HQ changes. The key nodes influencing SDR included pre, k, R, and slope. Slope had the highest VR proportion, indicating the most significant impact on SDR changes. The key nodes influencing CS included NDVI, k, POP, temperature (tem), actual evapotranspiration (AET), and land use. NDVI had the highest VR proportion, indicating the most significant impact on CS changes. The key nodes influencing WY included R, pre, tem and AET. R had the highest VR proportion, indicating the most significant impact on WY changes. The key nodes influencing FS included POP, slope, NDVI, land use, and k. Slope had the highest VR proportion, indicating the most significant impact on FS changes. Therefore, tem, slope, land use, k, R, pre, POP, and NDVI were identified as eight key nodes influencing the seven ESs, laying the foundation for analyzing the driving mechanisms of trade-offs and synergies among ESs in the following sections.

#### 3.2.3 Scenario simulation analysis.

This study drew on previous research [13,43,44]. Based on the trade-off and synergy effects of ESs and node importance analysis, we set different state probability values of ESs nodes in the BBN-ESs model as distinct scenarios. Subsequently, we identified key factors related to the trade-off and synergy effects of ESs using the BBN probabilistic reasoning method. The detailed descriptions of the scenario simulation are shown below, see Tables 2–3. Following the scenario framework detailed in Section 2.3.3.3, we calculated the relative changes between the posterior and prior probabilities of eight key node states under each scenario. Following the procedure in Section 2.3.3.4, the 95th percentile of absolute probability changes was adopted as the threshold for identifying primary associated factors. The calculated threshold was 55.9% (95% bootstrap confidence interval: [49.63%, 62.50%]).

**Fig 4:**
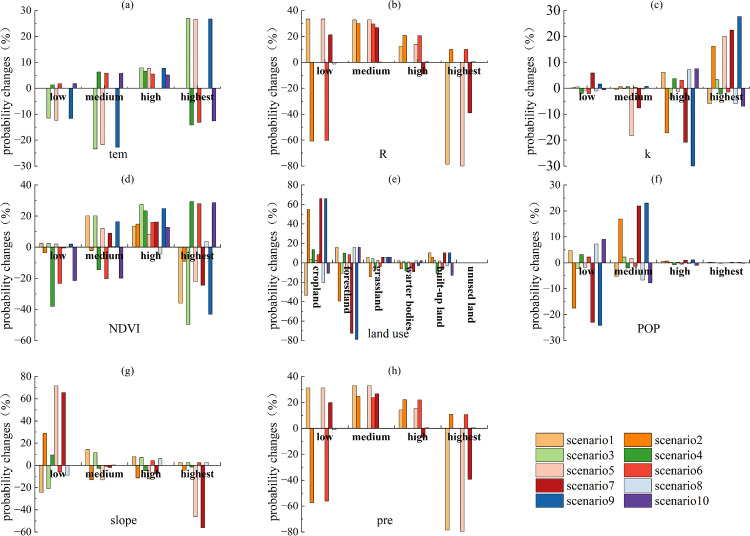
Probability changes of various factors in Scenario I (scenario1, scenario3, scenario5, scenario7, scenario9) and Scenario II (scenario2, scenario4, scenario6, scenario8, scenario10) in 2000.

**Fig 5:**
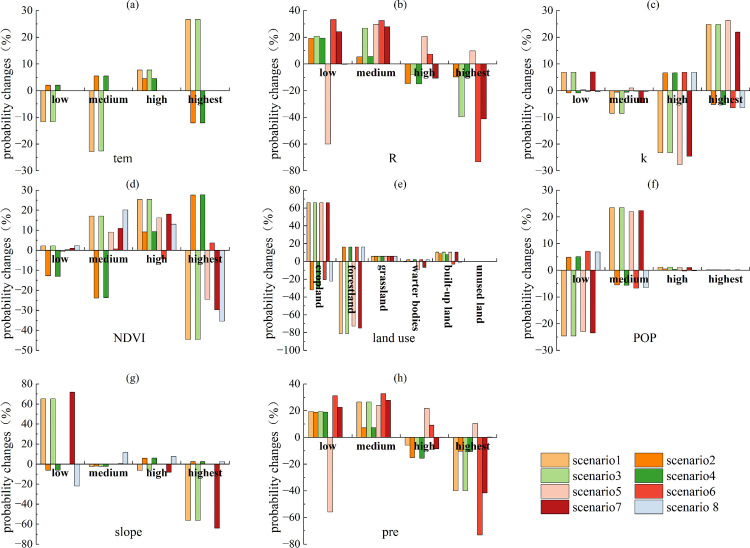
Probability changes of various factors in Scenario III (scenario1, scenario3, scenario5, scenario 7) and Scenario IV (scenario2, scenario4, scenario6, scenario8) in 2000.

As shown in Fig 4b, under Scenarios 2 and 6, the probability changes in the low states of R exceeded 55.9%; under Scenarios 1 and 5, the probability changes in the highest states of R exceeded 55.9%. Thus, R was identified as a factor with notable associations with ESs synergies. Under Scenarios 7 and 9, the probability changes of cropland and forestland in land use exceeded 55.9%. Thus, land use was identified as a factor with notable associations with ESs synergies. Under Scenarios 5, the probability changes in the low states of slope exceeded 55.9%; under Scenarios 7, the probability changes in the both the low states and the highest states of slope exceeded 55.9%. Thus, slope was identified as a factor strongly linked to ESs synergies. Similarly, under Scenarios 2 and 6, the probability changes in the low states of pre exceeded 55.9%; under Scenarios 1 and 5, the probability changes in the highest states of pre exceeded 55.9%. Thus, pre was identified as a factor showing associations with ESs synergies. Slope, land use, pre, and R all showed notable associations with synergies. Among these, land use was most notably associated with ESs synergies, followed by R and pre, and finally by slope. As shown in Fig 5, similarly, slope, land use, pre, and R all showed notable associations with trade-offs. Among these, land use was most notably associated with ESs trade-offs, followed by slope and R, and finally by pre.

**Fig 6:**
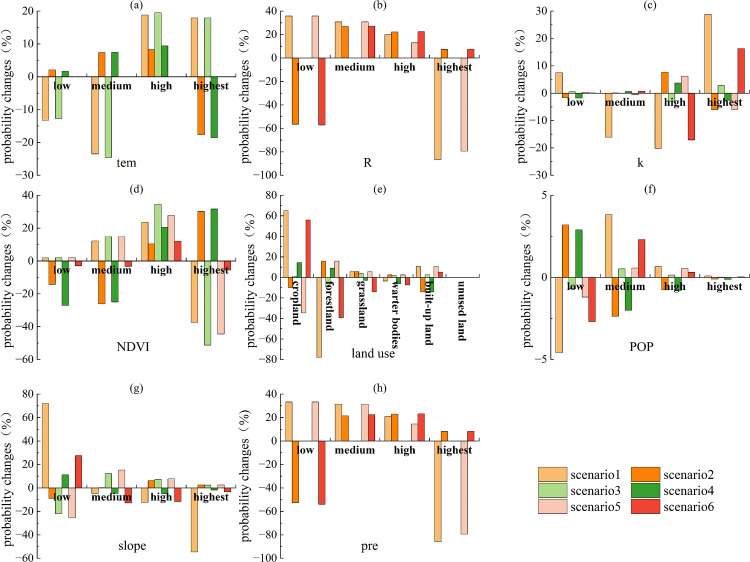
Probability changes of various factors in Scenario I (scenario1, scenario3, scenario5) and Scenario II (scenario2, scenario4, scenario6) in 2010.

**Fig 7:**
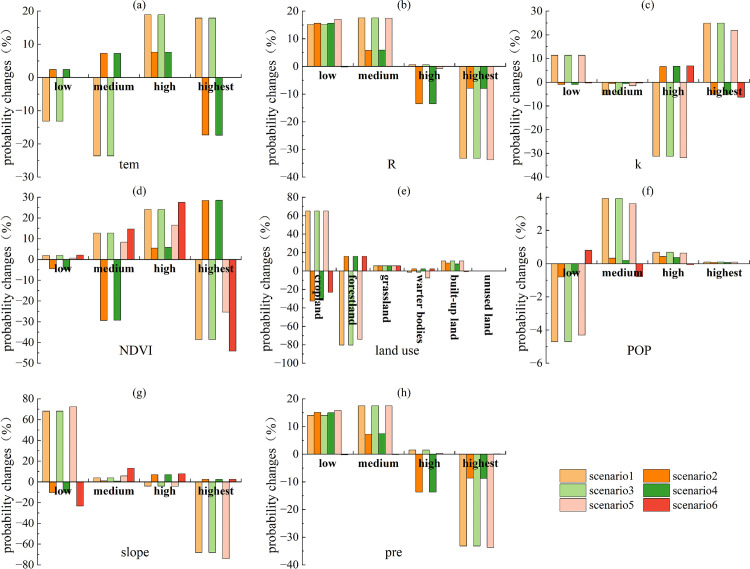
Probability changes of various factors in Scenario III (scenario1, scenario3, scenario5) and Scenario IV (scenario2, scenario4, scenario6) in 2010.

Based on the above analysis and in combination with Figs 6–7, the factors associated with ESs trade-offs and synergies in 2010 included slope, pre, land use, and R. R, slope, pre, and land use were the key factors notably associated with synergies (Fig 6). Among these, R was most notably associated with ESs synergies, followed by land use and pre, and finally by slope. Slope and land use were the key factors notably associated with trade-offs (Fig 7). Among these, land use was most notably associated with ESs trade-offs, followed by slope.

**Fig 8:**
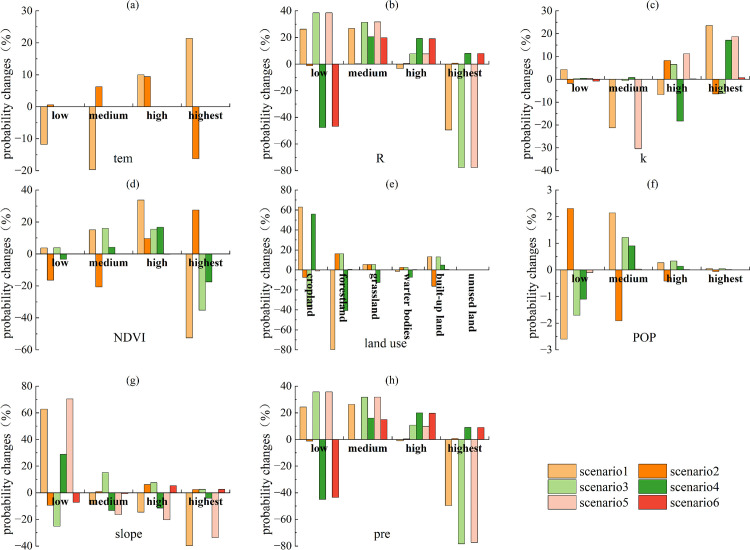
Probability changes of various factors in Scenario I (scenario1, scenario3, scenario5, scenario7) and Scenario II (scenario2, scenario4, scenario6, scenario8) in 2020.

**Fig 9:**
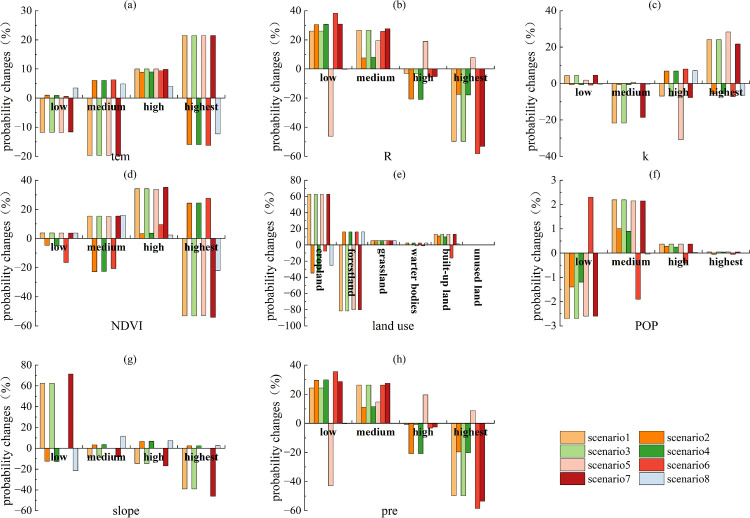
Probability changes of various factors in Scenario III (scenario1, scenario3, scenario5, scenario7) and Scenario IV (scenario2, scenario4, scenario6, scenario8) in 2020.

Based on the above analysis and in combination with Figs 8–9, the factors associated with ESs trade-offs and synergies in 2020 included slope, pre, land use, and R. R, slope, pre, and land use were the key factors notably associated with synergies (Fig 8). Among these, pre was most notably associated with ESs synergies, followed by R and land use, and finally by slope. R, slope, pre, and land use were the key factors notably associated with trade-offs (Fig 9). Among these, land use was most notably associated with ESs trade-offs, followed by slope and pre, and finally by R.

### 3.3 Threshold effects of key factors for trade-off/synergy relationships among ESs

**Fig 10:**
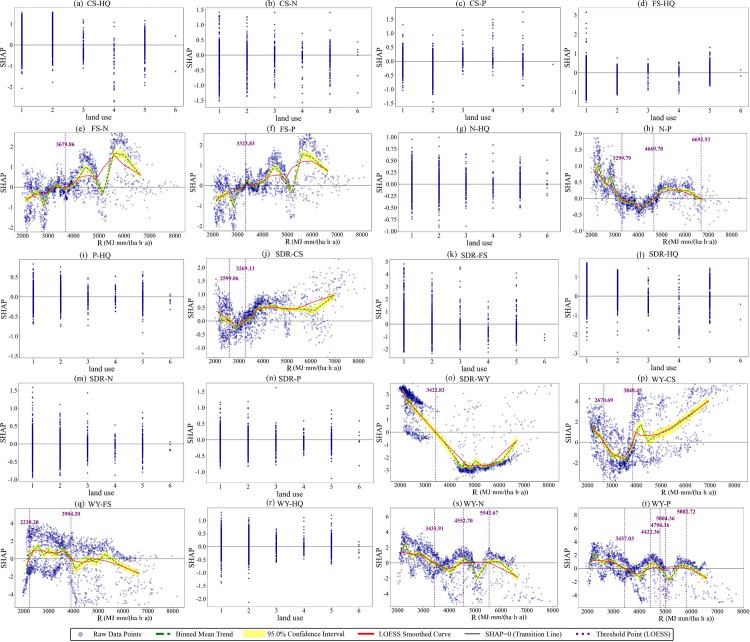
SHAP dependence plots for the factors associated with ESs trade-offs and synergies in 2000.

**Fig 11:**
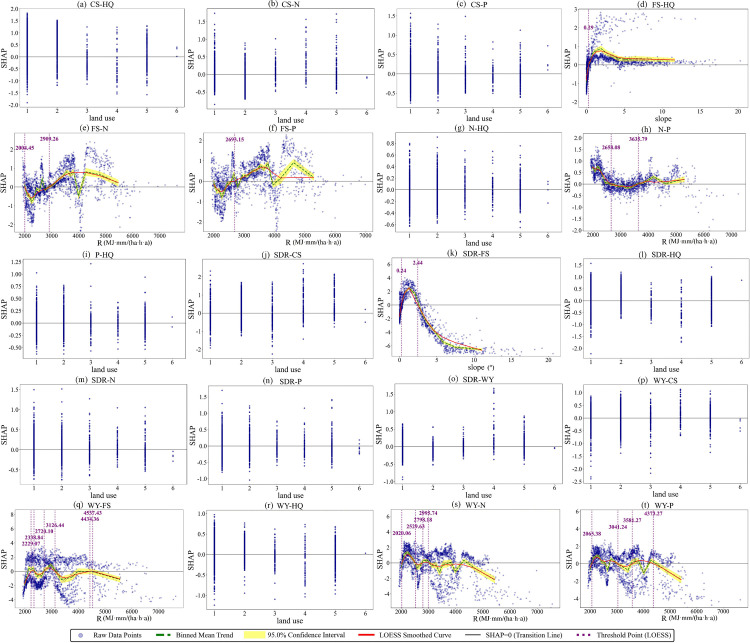
SHAP dependence plots for the factors associated with ESs trade-offs and synergies in 2010.

**Fig 12:**
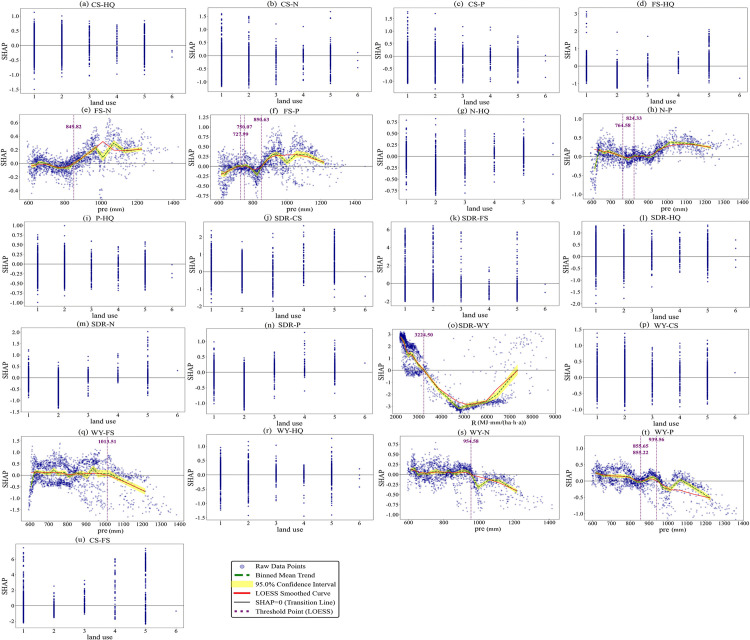
SHAP dependence plots for the factors associated with ESs trade-offs and synergies in 2020.

Based on multi-scenario analysis with the BBN-ESs model, this study identified the most critical influencing factors for each ESs pair. SHAP dependence plots were used to visualize and interpret the association patterns between these key factors and their corresponding ESs pairs (Figs 10–12). Land use types were coded as follows: 1 for Cropland, 2 for Forestland, 3 for Grassland, 4 for Water Bodies, 5 for Built-up Land, and 6 for Unused Land. In terms of analytical scope, 20 ESs pairs were analyzed for 2000 and 2010, as no significant influencing factor was identified for the FS-CS. All 21 ESs pairs were analyzed for 2020. Model accuracy was evaluated using the AUC, accuracy, F1-score, and per-class F1-scores. Complete parameter settings and accuracy evaluation results are provided in S6–S11 Tables (S4 Appendix).

As revealed by Fig 10, with the exception of unused land, all other land use types exhibited functional differentiation, showing differential positive associations with either synergies or trade-offs of specific ESs pairs; Unused Land had SHAP values consistently near zero due to its limited ecological function and small sample size, indicating no significant associations with either synergies or trade-offs (Fig 10). The relationship between R and ESs trade-offs/synergies exhibits a pattern of two discontinuous sensitive intervals, with the 2200–2700 MJ·mm/(ha·h·a) and 3400–4000 MJ·mm/(ha·h·a) ranges serving as sensitive intervals where directional shifts occur in their associations. Within the 2200–2700 MJ·mm/(ha·h·a) interval, the association of R with SDR-CS and WY-CS shifts from a positive association with synergy to a positive association with trade-off (Fig 10j, 10p), while its association with WY-FS shifts from trade-off to synergy (Fig 10q). In the 3400–4000 MJ·mm/(ha·h·a) interval, R shifts from a positive association with trade-offs to a positive association with synergies for FS-N, FS-P, SDR-CS, and WY-CS (Fig 10e, 10f, 10j, 10p). In contrast, it shifts from synergy to trade-off for N-P, SDR-WY, WY-FS, WY-N, and WY-P (Fig 10h, 10o, 10q, 10s, 10t). ESs related to N, P, and WY are more sensitive to R, as indicated by their higher number of thresholds.

As revealed by Fig 11, except for unused land, all land use types generally showed differential positive associations with ESs trade-offs and synergies; unused land had SHAP values close to zero due to weak ecological functions, with no significant associations (Fig 11). R and slope exhibit clear threshold characteristics. Specifically, R values in the 2000–3600 MJ·mm/(ha·h·a) and 4300–4500 MJ·mm/(ha·h·a) intervals represent sensitive ranges where directional reversals occur in these associations. For example, the relationship between R and the FS-N, FS-P, N-P, WY-FS, WY-N, and WY-P pairs undergoes a directional shift within the 2000–3600 MJ·mm/(ha·h·a) interval, that is, R changes from being positively associated with synergy to being positively associated with trade-off, or vice versa (Fig 11e, 11f, 11h, 11q, 11s, 11t). Similarly, the WY-FS and WY-P pairs also experience such a directional reversal within the 4300–4500 MJ·mm/(ha·h·a) interval (Fig 11q, 11t). In contrast, slope shows fewer threshold points in its relationship with ESs pairs. Only one threshold is identified for the FS-HQ pair, at 0.29°. When the slope exceeds this value, its association with FS-HQ shifts from synergy to trade-off (Fig 11d). For the SDR-FS pair, two threshold points are observed, and within the 0.24–2.44° range, slope maintains a positive association with synergy (Fig 11k).

As revealed by Fig 12, with the exception of unused land, all other land use types exhibited differential positive associations with synergies or trade-offs of distinct ESs pairs (Fig 12). Both R and pre exhibit clear threshold characteristics. A single threshold is identified for R in its relationship with SDR-WY at 3244.9 MJ·mm/(ha·h·a). When R exceeds this value, its association shifts from synergy to trade-off (Fig 12o). For precipitation, the 850–1000 mm range is a sensitive interval where directional reversals occur. For instance, the relationship between pre and the FS-N, FS-P, N-P, WY-FS, WY-N, and WY-P pairs undergoes a directional shift within this interval, that is, pre changes from being positively associated with synergy to being positively associated with trade-off, or vice versa (Fig 12e, 12f, 12h, 12q, 12s, 12t).

## 4 Discussion

### 4.1 Differentiated analysis of research on ESs trade-offs and synergies

Existing studies on the trade-offs and synergies of ESs in Henan Province and its subregions acknowledge the core regulatory roles of land use, topographic, and climatic factors [13,34,35], which is consistent with the four key factors (slope, land use, precipitation, and rainfall erosion) identified in this study. However, most studies have either focused on the driving mechanisms in a single time period [13] or emphasized the sole dominant role of land use [36,45], failing to reveal the temporal differentiation characteristics and the dominant effects of hydrological and topographic factors in specific time periods. More importantly, existing studies [14,42] have not addressed the accurate quantification of thresholds for key driving factors. In contrast, this study has systematically identified the sensitive intervals and thresholds of rainfall erosion (R), slope and precipitation, as well as the functional differentiation characteristics of different land use types via the XGBoost-SHAP model.

The average accuracy of the BBN-ESs model exceeds 70%, which indicates that the model can effectively capture the dynamic changes of ESs and thus provides support for exploring the relevant influencing factors of their trade-offs and synergies. However, it should be noted that there are differences in the model accuracy among different types of ESs: SDR and HQ have the highest accuracy, while the accuracy of N and P indicators is relatively low. The reason for the lower accuracy of N and P indicators may be that some driving variables are not included in the model [46].

Based on the BBN-ESs model and a 55.9% statistical threshold, this study identified slope, pre, land use, and R as the key factors influencing ESs trade-offs and synergies in Henan Province. These factors together form the basic framework for regional ESs interactions. The framework shows that natural factors such as R and pre have strong statistical associations with ESs like WY and SDR, which is consistent with the conclusions of Yuan et al.[47] and Feng et al.[48]. Notably, in the synergy scenarios of 2010 and 2020, the association strength of R and pre exceeded that of land use, which differs from the view of Chen et al.[45] who emphasized land use as the dominant driving factor. This difference is mainly attributed to two aspects: Henan’s terrain, higher in the west and lower in the east, has strengthened the association between hydrological processes and ESs; methodologically, the BBN model can more effectively reveal the nonlinear relationships and conditional dependencies among multiple factors [49]. It should be noted that the conclusions of this study are statistical associations based on observational data, which can provide hypotheses for subsequent causal research.

### 4.2 Potential mechanisms and theoretical rationale of ESs relationship shifts

This study used SHAP analysis to identify the critical thresholds driving the state transitions of ESs trade-offs and synergies, and this section discusses their driving mechanisms and theoretical rationale. The threshold transitions of ESs relationships are jointly driven by hydrology, topography, and land use, which can be explained by regime shift theory and tipping point theory. When precipitation exceeds approximately 1000 mm, the soil-vegetation system reaches water saturation state. This not only fails to support crop growth but may also cause flooding and nutrient loss, shifting the relationship between WY and FS from synergy to trade-off [50]. This transition is consistent with the characteristics of regime shift, where the system undergoes abrupt changes between stable states [51]. When R exceeds about 3422 MJ·mm/(ha·h·a), the surface hydrological process undergoes a fundamental transformation, shifting from being dominated by rainfall infiltration to severe erosion, leading to a direct conflict between SDR and WY [52–53]. When the slope exceeds 2.44°, gravitational forces make traditional farming incompatible with soil and water conservation. Ecological restoration is often required through converting cropland to forest or grassland [54–55], thus forming a trade-off relationship between SDR and FS. These quantified thresholds support the core view of tipping point theory, which emphasizes that complex systems tend to undergo abrupt changes near critical points [56]. As a categorical variable, land use determines the basic pattern of ESs relationships through its ecological functions. For example, forestland usually forms synergistic relationships with CS and HQ, while cropland typically leads to trade-offs between FS and SDR, N/P, etc.[57]

### 4.3 Regulatory measures and potential risks based on the associative characteristics of ESs trade-offs and synergies

Combining the BBN-ESs model and SHAP analysis, this study identified the key factors and their threshold characteristics associated with ESs trade-offs and synergies. Notably, all conclusions are based on correlational analysis rather than causal verification, and the proposed management recommendations require field validation. Based on this, a management hypothesis of “zone-specific standard setting and category-based regulation” is put forward: (1) In the eastern plain agricultural areas, the planting structure should be optimized to maintain the synergistic relationships between ESs such as CS and HQ [57]. (2) In areas where R is below 2200 MJ·mm/(ha·h·a), measures including water supplementation, soil moisture conservation, and vegetation optimization should be adopted; in areas where R exceeds 3000 MJ·mm/(ha·h·a), erosion control should be implemented through vegetation interception and engineering measures [52–53]. (3) For croplands with a slope exceeding 2.44°, engineering modification and crop adaptation should be carried out in areas where cultivation continues; in severely eroded areas, graded conversion of cropland to forest/grassland and composite vegetation management should be implemented [54–55]. (4) In regions with precipitation exceeding 850 mm, vegetation coverage should be optimized to enhance the synergistic effects between FS and N/P; in regions with precipitation exceeding 1000 mm, the trade-off relationship between WY and FS should be alleviated by improving drainage systems and adjusting vegetation structures [50].

These regulatory measures may face several potential risks: First, vegetation adjustments may compete with native crops and local species for light, water, and nutrient resources, leading to resource competition and adaptation issues. Second, engineering measures require high initial investment, and water supplementation schemes rely on stable water sources, posing economic cost and sustainability challenges. Third, engineering modification and cropland conversion may temporarily reduce local grain yields, making it still challenging to balance food security and ecological protection.

### 4.4 Limitations and prospects

This study provides an in-depth exploration of the key factors and threshold effects associated with ES trade-offs and synergies. However, several limitations in the research process should be acknowledged. First, limitations in the parameter settings for ES quantification resulted in discrepancies between simulated and actual values. Second, this study employed a BBN to investigate the underlying relationships behind trade-offs and synergies among ESs. This method effectively captures nonlinear statistical associations within the research data, providing robust support for analyzing the interconnections among ESs. This study’s analysis identifies strong statistical associations, which offer robust hypotheses and insights into potential causal mechanisms. Yet, as this associational analysis is based on observational data, it is insufficient to definitively establish confirmed causation. However, due to its inherent characteristics, the BBN analysis was confined to the grid scale and could not be extended to broader administrative scales, such as municipal or county levels. Future research should adopt targeted improvements. First, ground-based monitoring should be conducted to calibrate parameters in the InVEST model, thereby enhancing its accuracy. Furthermore, integrating complementary methodologies or refining the BBN framework could help overcome its scale limitations, enabling a multi-scale exploration of these associational patterns.

## 5 Conclusions

This study takes Henan Province as the study area. Based on multi-source data from 2000 to 2020, the InVEST model, BBN, and XGBoost-SHAP model were integrated to systematically analyze the spatio-temporal evolution patterns of trade-offs/synergies among seven key ESs, identify the core driving factors underlying changes in service relationships, and quantify the threshold effects of key factors. Three core conclusions are finally drawn: (1) ESs in Henan Province exhibited coexisting synergies and trade-offs from 2000 to 2020; (2) Slope, precipitation, land use, and rainfall erosion were the four core driving factors during the study period, and the influence intensity of hydrological and topographic factors on service relationships exceeded that of land use in 2010 and 2020; (3) Distinct functional differentiation characteristics were observed in the trade-offs/synergies of ESs across different land use types, and significant nonlinear threshold effects existed for continuous driving factors such as rainfall erosion, precipitation, and slope.

Based on the above analysis, this study challenges the conventional understanding in similar studies that “land use is the sole dominant driving factor of ecosystem services,” and confirms the core role of hydrological and topographic factors in the evolution of ESs in major grain-producing areas. The established analytical framework of “BBN for driving factor identification and XGBoost-SHAP for threshold quantification” fills the research gap in the refined investigation of nonlinear driving mechanisms and threshold effects of ESs in Henan Province. Meanwhile, the identified functional differentiation patterns of ESs across different land use types, as well as the sensitive intervals of rainfall erosion (2200–2700 MJ·mm/(ha·h·a)) and precipitation (850–1000 mm), together with the critical threshold of slope (2.44°), provide a quantitative scientific basis for ecological environment management in Henan Province. The zonal management strategy formed on the basis of the four core driving factors can accommodate the differentiated ecological governance demands of the eastern plain agricultural areas and the western mountainous and hilly regions of Henan Province.

This study still has certain limitations. In the future, the measured data from ground ecological monitoring stations in Henan Province can be integrated to calibrate the localized parameters of the InVEST model, so as to further improve the quantification accuracy of ESs. The analysis scale of the driving mechanism can be expanded from grids to municipal and county administrative units to realize multi-scale correlation analysis, enabling the research results to better support the formulation of local ecological management policies. Overall, this study systematically clarifies the interaction rules and driving mechanisms of ESs in Henan Province. The research results can not only provide a scientific basis for local ecological governance in Henan Province, but also offer a reference for the refined management of ecosystems in other major grain-producing areas in China and similar regions in the middle and lower reaches of the Yellow River Basin.

## Supporting information

S1 Appendix 95% confidence intervals and false discovery rate-adjusted p-value. (DOCX)

S2 Appendix Construction of the Bayesian belief network-ecosystem services model. (DOCX)

S3 Appendix 2000–2020 BBN-ESs model accuracy figures and factor nodes importance analysis. (DOCX)

S4 Appendix Optimized XGBoost parameters and accuracy verification. (DOCX)

S1 Fig The BBN-ESs model. (TIF)

S2 Fig The accuracy of the BBN-ESs Model in 2000, 2010, and 2020. (TIF)

S3 Fig Analysis of the importance of factor nodes to ESs nodes in 2000, 2010, and 2020. (TIF)

S1 Table 95% Confidence intervals for each service type. (DOCX)

S2 Table p-values and FDR-adjusted p-values for each service type (DOCX)

S3 Table State classification and range division of each node in the BBN-ESs Model in 2000. (DOCX)

S4 Table State classification and range division of each node in the BBN-ESs Model in 2010. (DOCX)

S5 Table State classification and range division of each node in the BBN-ESs Model in 2020. (DOCX)

S6 Table Optimized parameters for the XGBoost model (2000). (DOCX)

S7 Table Optimized parameters for the XGBoost model (2010). (DOCX)

S8 Table Optimized parameters for the XGBoost model (2020). (DOCX)

S9 Table Accuracy of the XGBoost Model on the Test Set (2000). (DOCX)

S10 Table Accuracy of the XGBoost model on the test set (2010). (DOCX)

S11 Table Accuracy of the XGBoost model on the test set (2020). (DOCX)
