# Supplementary material for: Identifying driving mechanisms and threshold effects of trade-offs and synergies among ecosystem services: A case study of Henan Province, China
Source: PLoS One. 2026 Apr 21;21(4):e0347200. doi: 10.1371/journal.pone.0347200 (PMC13099101; doi:10.1371/journal.pone.0347200)
Supplement: S1 Appendix — (DOCX) [file pone.0347200.s001.docx]

# S1 Appendix: 95% confidence intervals and False Discovery Rate-adjusted p-values

S1 Table 1. 95% Confidence Intervals for Each Service Type

|  | 2000 | | 2010 | | 2020 | |
| --- | --- | --- | --- | --- | --- | --- |
| Types | Lower Limit | Upper Limit | Lower Limit | Upper Limit | Lower Limit | Upper Limit |
| HQ-SDR | 0.531 | 0.571 | 0.550 | 1.000 | 0.478 | 0.521 |
| HQ-CS | 0.503 | 0.544 | 0.500 | 1.000 | 0.562 | 0.600 |
| HQ-FS | -0.325 | -0.273 | -1.000 | -0.314 | -0.256 | -0.202 |
| HQ-N | -0.313 | -0.260 | -1.000 | -0.328 | -0.346 | -0.295 |
| HQ-P | -0.318 | -0.266 | -1.000 | -0.336 | -0.355 | -0.304 |
| HQ-WY | -0.103 | -0.046 | -0.014 | 1.000 | -0.247 | -0.192 |
| SDR-CS | 0.365 | 0.413 | 0.344 | 1.000 | 0.344 | 0.394 |
| SDR-FS | -0.450 | -0.404 | -1.000 | -0.462 | -0.417 | -0.369 |
| SDR-N | -0.178 | -0.122 | -1.000 | -0.197 | -0.257 | -0.203 |
| SDR-P | -0.178 | -0.122 | -1.000 | -0.199 | -0.259 | -0.205 |
| SDR-WY | 0.159 | 0.215 | 0.230 | 1.000 | 0.077 | 0.133 |
| CS-FS | 0.006 | 0.063 | 0.000 | 1.000 | -0.037 | 0.020 |
| CS-N | -0.143 | -.086 | -1.000 | -0.117 | -0.128 | -0.072 |
| CS-P | -0.158 | -0.102 | -1.000 | -0.137 | -0.153 | -0.097 |
| CS-WY | 0.074 | 0.130 | 0.118 | 1.000 | -0.169 | -0.113 |
| FS-N | 0.342 | 0.391 | 0.352 | 1.000 | 0.404 | 0.450 |
| FS-P | 0.337 | 0.386 | 0.347 | 1.000 | 0.398 | 0.445 |
| FS-WY | 0.068 | 0.125 | -0.021 | 1.000 | 0.008 | 0.065 |
| N-P | 0.999 | 0.999 | 0.999 | 1.000 | 0.999 | 0.999 |
| N-WY | 0.285 | 0.337 | 0.175 | 1.000 | 0.232 | 0.285 |
| P-WY | 0.286 | 0.338 | 0.175 | 1.000 | 0.237 | 0.291 |

S1 Table 2. p-values and FDR-adjusted p-values for each service type

|  | 2000 | | 2010 | | 2020 | |
| --- | --- | --- | --- | --- | --- | --- |
| Types | p-values | FDR-adjusted p-values | p-values | FDR-adjusted p-values | p-values | FDR-adjusted p-values |
| HQ-SDR | 0.000 | 0.000 | 0.000 | 0.000 | 0.000 | 0.000 |
| HQ-CS | 0.000 | 0.000 | 0.000 | 0.000 | 0.000 | 0.000 |
| HQ-FS | <0.001 | 0.00525 | <0.001 | 0.00525 | <0.001 | 0.00525 |
| HQ-N | <0.001 | 0.00525 | <0.001 | 0.00525 | <0.001 | 0.00525 |
| HQ-P | <0.001 | 0.00525 | <0.001 | 0.00525 | <0.001 | 0.00525 |
| HQ-WY | <0.001 | 0.00525 | 0.234 | 0.2457 | <0.001 | 0.00525 |
| SDR-CS | <0.001 | 0.00525 | <0.001 | 0.00525 | <0.001 | 0.00525 |
| SDR-FS | <0.001 | 0.00525 | <0.001 | 0.00525 | <0.001 | 0.00525 |
| SDR-N | <0.001 | 0.00525 | <0.001 | 0.00525 | <0.001 | 0.00525 |
| SDR-P | <0.001 | 0.00525 | <0.001 | 0.00525 | <0.001 | 0.00525 |
| SDR-WY | <0.001 | 0.00525 | <0.001 | 0.00525 | <0.001 | 0.00525 |
| CS-FS | 0.014 | 0.014 | 0.044 | 0.0486 | 0.537 | 0.537 |
| CS-N | <0.001 | 0.00525 | <0.001 | 0.00525 | <0.001 | 0.00525 |
| CS-P | <0.001 | 0.00525 | <0.001 | 0.00525 | <0.001 | 0.00525 |
| CS-WY | <0.001 | 0.00525 | <0.001 | 0.00525 | <0.001 | 0.00525 |
| FS-N | <0.001 | 0.00525 | <0.001 | 0.00525 | <0.001 | 0.00525 |
| FS-P | <0.001 | 0.00525 | <0.001 | 0.00525 | <0.001 | 0.00525 |
| FS-WY | <0.001 | 0.00525 | 0.426 | 0.426 | 0.010 | 0.0105 |
| N-P | 0.000 | 0.000 | 0.000 | 0.000 | 0.000 | 0.000 |
| N-WY | <0.001 | 0.00525 | <0.001 | 0.00525 | <0.001 | 0.00525 |
| P-WY | <0.001 | 0.00525 | <0.001 | 0.00525 | <0.001 | 0.00525 |
