# Supplementary material for: Identifying driving mechanisms and threshold effects of trade-offs and synergies among ecosystem services: A case study of Henan Province, China
Source: PLoS One. 2026 Apr 21;21(4):e0347200. doi: 10.1371/journal.pone.0347200 (PMC13099101; doi:10.1371/journal.pone.0347200)
Supplement: S2 Appendix — (DOCX) [file pone.0347200.s002.docx]

# S2 Appendix: Construction of the Bayesian Belief Network-Ecosystem Services Model

This study uses Netica software to construct the Bayesian Belief Network-Ecosystem Services (BBN-ESs) model. The specific construction process is divided into the following steps: First, the seven types of ESs focused on in this study (i.e., SDR, N, P, CS, FS, WY, HQ) were designated as the target nodes of the model, which directly correspond to the types of ecosystem services to be evaluated in the study. Subsequently, based on the data required for the simulation of ESs, and considering the data availability and data quality in the study area, factors including population, slope, precipitation, land use, temperature, Normalized Difference Vegetation Index (NDVI), soil erosion, rainfall erosion (R), and actual evapotranspiration (AET) were selected as the driver nodes of the model. This selection aims to accurately reflect the key factors influencing the target ESs. Meanwhile, by referring to the achievements of scholars such as Sun et al.^[1]^, Li et al.^[2]^, and Shen et al^[3]^. (in the field of exploring the driving mechanisms of ecosystem services using Bayesian Belief Networks, the connection relationships between the driver nodes and target nodes (i.e., the “edges” of the model) were further verified and clarified. This ensures that the associations between nodes are consistent with the existing research consensus and the actual characteristics of the study area. Next, the data layers for each node were discretized using ArcGIS software. The land use node, as a discrete variable, was classified into six categories based on the Chinese Academy of Sciences land use and land cover classification system. The other nodes were continuous variables, which were divided into four states: low, medium, high, and highest using the natural break-point method. Specific variable discretization criteria are shown in S2 Tables 1–3. A 1 km×1 km grid was created to extract the raster values from each layer, and parameter learning was conducted for the BBN. Finally, the extracted sample data were input into the network for model training, and conditional probability tables (CPTs) for all nodes of the BBN were obtained, completing the construction of the BBN-ESs model (S2 Fig 1).


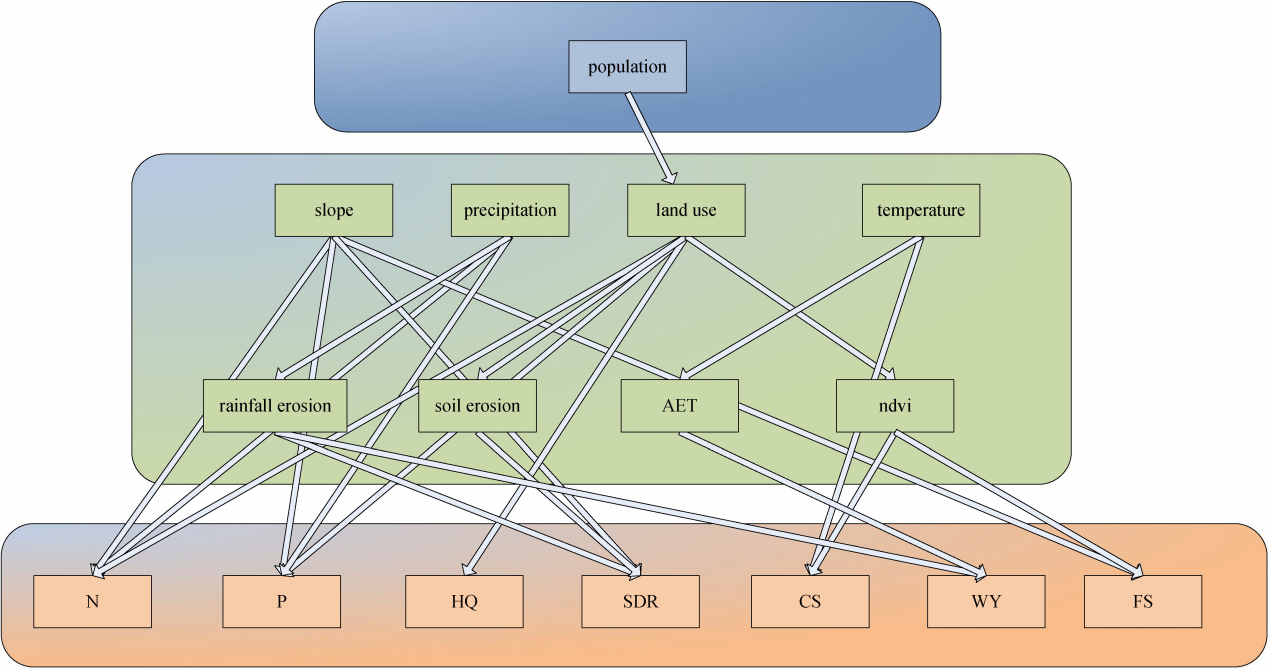


S2 Fig 1. The BBN-ESs model

S2 Table 1. State classification and range division of each node in the BBN-ESs Model in 2000

| Nodes | State and scope（2000） | | | | Unit |
| --- | --- | --- | --- | --- | --- |
|  | low | medium | high | highest |  |
| Population | [0,606) | [606,3522) | [3522,12152) | [12152,47777] | People/km^2^ |
| Slope | [0,1.4) | [1.44,4.2) | [4.2,8.5) | [8.5,24.4] | ° |
| Precipitation | [549.6,708.4) | [708.4,860.5) | [860.9,1027.8) | [1027.8,1478.3] | mm |
| Land Use | cropland, forestland, grassland, waters, building, others | | | | — |
| Temperature | [5.4,11.8) | [11.8,13.8) | [13.8,15.1) | [15.1,16.6] | ℃ |
| Rainfall erosion | [1913.5,2965.1) | [2965.1,4061.7) | [4061.7,5388.8) | [5388.8,9665.6] | MJ·mm/(ha·h·a) |
| Soil erosion | [0.0.010) | [0.010,0.014) | [0.014,0.018) | [0.018,0.021] | t·ha·h/(ha·MJ·mm) |
| AET | [720.2,1030.6) | [1030.6,1138.4) | [1038.4,1200.4) | [1200.4,1269.0] | mm |
| NDVI | [0,0.5) | [0.5,0.6) | [0.6,0.7) | [0.7,1] | — |
| P | [0,30.3) | [30.3,75.2) | [75.2,120.1) | [120.1,287.4] | kg |
| N | [0,192.6) | [192.6,467.7) | [467.7,742.9) | [724.9,1761.1] | kg |
| HQ | [0,0.4) | [0.4,0.6) | [0.6,0.8) | [0.8,1] | — |
| SDR | [0,27.0) | [27.0,102.6) | [102.6,275.5) | [275.5,1382.8] | kt |
| CS | [0,3640) | [3640,6776) | [6776,8404) | [8404,13353] | t |
| WY | [0,295.4) | [295.4,437.1) | [437.1,598.9) | [598.9,1036.1] | mm |
| FS | [0,151.7) | [151.7,338.1) | [338.1,387.1) | [387.1,525.7] | t |

S2 Table 2. State classification and range division of each node in the BBN-ESs Model in 2010

| Nodes | State and scope（2010） | | | | Unit |
| --- | --- | --- | --- | --- | --- |
|  | low | medium | high | highest |  |
| Population | [0,1393) | [1393,5471) | [5472,16703) | [16703,65451] | People/km^2^ |
| Slope | [0,1.4) | [1.44,4.2) | [4.2,8.5) | [8.5,24.4] | ° |
| Precipitation | [547.6,639.3) | [639.3,749.7) | [749.7,900.7) | [900.7,1350.1] | mm |
| Land Use | cropland, forestland, grassland, waters, building, others | | | | — |
| Temperature | [5.6,12.1) | [12.1,14.1) | [14.1,15.3) | [15.3,16.8] | ℃ |
| Rainfall erosion | [1901.7,2488.4) | [2488.4,3236.2) | [3236.2,4356.7) | [4356.7,8253.3] | MJ·mm/(ha·h·a) |
| Soil erosion | [0.0.010) | [0.010,0.014) | [0.014,0.018) | [0.018,0.021] | t·ha·h/(ha·MJ·mm) |
| AET | [722.7,1022.5) | [1022.5,1127.1) | [1127.1,1186.3) | [1186.3,1267.2] | mm |
| NDVI | [0,0.5) | [0.5,0.7) | [0.7,0.8) | [0.8,1] | — |
| P | [0,32.1) | [32.1,77.4) | [77.4,122.8) | [122.8,283.4] | kg |
| N | [0,196.7) | [196.7,474.8) | [474.8,753.1) | [753.1,1736.7] | kg |
| HQ | [0,0.4) | [0.4,0.7) | [0.7,0.8) | [0.9,1] | — |
| SDR | [0,17.9) | [17.9,71.6) | [71.6,175.4) | [175.4,916.3] | kt |
| CS | [0,3640) | [3640,6776) | [6776,8404) | [8404,13353] | t |
| WY | [0,243.1) | [243.1,346.8) | [346.8,489.8) | [489.8,915.4] | mm |
| FS | [0,206.9) | [206.9,455.7) | [455.7,516.9) | [516.9,646.5] | t |

S2 Table 3. State classification and range division of each node in the BBN-ESs Model in 2020

| Nodes | State and scope（2020） | | | | Unit |
| --- | --- | --- | --- | --- | --- |
|  | low | medium | high | highest |  |
| Population | [0,2183) | [2183,8694) | [8694,25372) | [25372,78458] | People/km^2^ |
| Slope | [0,1.4) | [1.44,4.2) | [4.2,8.5) | [8.5,24.4] | ° |
| Precipitation | [590.7,719.4) | [719.4,863.5) | [863.5,1043.5) | [1043.5,1552.5] | mm |
| Land Use | cropland, forestland, grassland, waters, building, others | | | | — |
| Temperature | [5.7,12.4) | [12.4,14.5) | [14.5,15.8) | [15.8,17.1] | ℃ |
| Rainfall Erosion | [2151.3,3042.6) | [3042.6,4111.3) | [4111.3,5578.6) | [5578.6,10359.4] | MJ·mm/(ha·h·a) |
| Soil Erosion | [0.0.010) | [0.010,0.014) | [0.014,0.018) | [0.018,0.021] | t·ha·h/(ha·MJ·mm) |
| AET | [726.1,1031.3) | [1031.3,1139.1) | [1139.1,1214.3) | [1214.3,1290.2] | mm |
| NDVI | [0,0.5) | [0.5,0.7) | [0.7,0.8) | [0.8,1] | — |
| P | [0,30.1) | [30.1,75.2) | [75.2,124.2) | [124.2,321.3] | kg |
| N | [0,184.5) | [184.5,461.4) | [461.4,769.1) | [769.1,1968.8] | kg |
| HQ | [0,0.4) | [0.4,0.6) | [0.6,0.8) | [0.8,1] | — |
| SDR | [0,25.4) | [25.4,111.6) | [111.6,334.9) | [334.9,1299.0] | kt |
| CS | [0,3640) | [3640,6776) | [6776,8404) | [8404,13353] | t |
| WY | [0,166.3) | [166.3,297.1) | [297.1,467.4) | [467.4,1014.1] | mm |
| FS | [0,266.8) | [266.8,587.7) | [587.7,673.3) | [673.3,847.9] | t |

References：

1. Sun Y, Lin X, Li S, Wang J, Luo C. 2024. Spatiotemporal dynamics and driving factor assessment of ecosystem service trade-offs in Guizhou Province [J]. Journal of Environmental Sciences, 44(09):401-413. https://doi.org/10.13671/j.hjkxxb.2024.0302.
2. Li J, Zhao W, Ma X, Luo G, Pereira P. 2025. Ecosystem service tradeoff and synergy mechanisms in the Central Asian terminal lake basin based on Bayesian Networks[J]. Ecosystem Services, 75, 101768-101768. https://doi.org/10.1016/j.ecoser.2025.101768.
3. Shen J, Zhao M, Tan Z, Zhu L, Guo Y, Li Y, et al. 2024. Ecosystem service trade-offs and synergies relationships and their driving factor analysis based on the Bayesian belief Network: A case study of the Yellow River Basin[J]. Ecological Indicators, 163, 112070. https://doi.org/10.1016/j.ecolind.2024.112070.
