# Supplementary material for: Identifying driving mechanisms and threshold effects of trade-offs and synergies among ecosystem services: A case study of Henan Province, China
Source: PLoS One. 2026 Apr 21;21(4):e0347200. doi: 10.1371/journal.pone.0347200 (PMC13099101; doi:10.1371/journal.pone.0347200)
Supplement: S3 Appendix — (DOCX) [file pone.0347200.s003.docx]

# S3 Appendix: 2000–2020 BBN-ESs Model Accuracy Figures and Factor Nodes Importance Analysis





S3 Fig 1. Analysis of the importance of factor nodes to ESs nodes in 2000, 2010, and 2020





S3 Fig 2. The accuracy of the BBN-ESs Model in 2000, 2010, and 2020
