# Supplementary material for: Identifying driving mechanisms and threshold effects of trade-offs and synergies among ecosystem services: A case study of Henan Province, China
Source: PLoS One. 2026 Apr 21;21(4):e0347200. doi: 10.1371/journal.pone.0347200 (PMC13099101; doi:10.1371/journal.pone.0347200)
Supplement: S4 Appendix — (DOCX) [file pone.0347200.s004.docx]

# S4 Appendix：Optimized XGBoost Parameters and Accuracy Verification

S4 Table 1. Optimized Parameters for the XGBoost Model (2000)

| Parameter Types | | | | | |
| --- | --- | --- | --- | --- | --- |
| Types | colsample_bytree | learning_rate | max_depth | n_estimators | subsample |
| CS-HQ | 0.8 | 0.15 | 7 | 150 | 0.8 |
| CS-N | 0.7 | 0.15 | 7 | 150 | 0.8 |
| CS-P | 0.7 | 0.15 | 7 | 150 | 0.7 |
| FS-HQ | 0.8 | 0.1 | 7 | 150 | 0.8 |
| FS-N | 0.8 | 0.15 | 7 | 150 | 0.7 |
| FS-P | 0.8 | 0.15 | 7 | 150 | 0.8 |
| N-HQ | 0.8 | 0.15 | 7 | 150 | 0.8 |
| N-P | 0.8 | 0.15 | 7 | 200 | 0.8 |
| P-HQ | 0.7 | 0.15 | 7 | 150 | 0.8 |
| SDR-CS | 0.8 | 0.15 | 7 | 150 | 0.8 |
| SDR-FS | 0.8 | 0.15 | 7 | 150 | 0.8 |
| SDR-HQ | 0.7 | 0.15 | 7 | 150 | 0.8 |
| SDR-N | 0.8 | 0.15 | 7 | 150 | 0.8 |
| SDR-P | 0.8 | 0.15 | 7 | 150 | 0.8 |
| SDR-WY | 0.8 | 0.4 | 6 | 150 | 0.8 |
| WY-CS | 0.8 | 0.15 | 7 | 150 | 0.7 |
| WY-FS | 0.8 | 0.15 | 7 | 150 | 0.8 |
| WY-HQ | 0.8 | 0.15 | 7 | 150 | 0.7 |
| WY-N | 0.8 | 0.15 | 7 | 150 | 0.8 |
| WY-P | 0.7 | 0.15 | 7 | 150 | 0.8 |

S4 Table 2. Optimized Parameters for the XGBoost Model (2010)

| Parameter Types | | | | | |
| --- | --- | --- | --- | --- | --- |
| Types | colsample_bytree | learning_rate | max_depth | n_estimators | subsample |
| CS-HQ | 0.8 | 0.15 | 7 | 150 | 0.8 |
| CS-N | 0.7 | 0.15 | 7 | 150 | 0.8 |
| CS-P | 0.7 | 0.15 | 7 | 150 | 0.7 |
| FS-HQ | 0.8 | 0.1 | 7 | 150 | 0.8 |
| FS-N | 0.8 | 0.15 | 7 | 150 | 0.7 |
| FS-P | 0.8 | 0.15 | 7 | 150 | 0.8 |
| N-HQ | 0.8 | 0.15 | 7 | 150 | 0.8 |
| N-P | 0.8 | 0.15 | 7 | 200 | 0.8 |
| P-HQ | 0.7 | 0.15 | 7 | 150 | 0.8 |
| SDR-CS | 0.8 | 0.15 | 7 | 150 | 0.8 |
| SDR-FS | 0.8 | 0.15 | 7 | 150 | 0.8 |
| SDR-HQ | 0.7 | 0.15 | 7 | 150 | 0.8 |
| SDR-N | 0.8 | 0.15 | 7 | 150 | 0.8 |
| SDR-P | 0.8 | 0.15 | 7 | 150 | 0.8 |
| SDR-WY | 0.8 | 0.1 | 6 | 150 | 0.8 |
| WY-CS | 0.8 | 0.15 | 7 | 150 | 0.7 |
| WY-FS | 0.8 | 0.15 | 7 | 150 | 0.8 |
| WY-HQ | 0.8 | 0.15 | 7 | 150 | 0.7 |
| WY-N | 0.8 | 0.15 | 7 | 150 | 0.8 |
| WY-P | 0.7 | 0.15 | 7 | 150 | 0.8 |

S4 Table 3. Optimized Parameters for the XGBoost Model (2020)

| Parameter Types | | | | | |
| --- | --- | --- | --- | --- | --- |
| Types | colsample_bytree | learning_rate | max_depth | n_estimators | subsample |
| CS-FS | 0.7 | 0.15 | 7 | 150 | 0.8 |
| CS-HQ | 0.7 | 0.1 | 7 | 150 | 0.7 |
| CS-N | 0.7 | 0.15 | 7 | 150 | 0.7 |
| CS-P | 0.8 | 0.15 | 7 | 150 | 0.8 |
| FS-HQ | 0.7 | 0.1 | 7 | 150 | 0.8 |
| FS-N | 0.8 | 0.15 | 7 | 150 | 0.8 |
| FS-P | 0.7 | 0.15 | 7 | 150 | 0.8 |
| N-HQ | 0.8 | 0.15 | 7 | 150 | 0.8 |
| N-P | 0.8 | 0.15 | 7 | 200 | 0.7 |
| P-HQ | 0.8 | 0.15 | 7 | 150 | 0.7 |
| SDR-CS | 0.8 | 0.15 | 7 | 150 | 0.8 |
| SDR-FS | 0.7 | 0.15 | 7 | 150 | 0.8 |
| SDR-HQ | 0.8 | 0.15 | 7 | 150 | 0.8 |
| SDR-N | 0.8 | 0.15 | 7 | 150 | 0.7 |
| SDR-P | 0.7 | 0.15 | 7 | 150 | 0.8 |
| SDR-WY | 0.7 | 0.1 | 7 | 150 | 0.8 |
| WY-CS | 0.8 | 0.1 | 7 | 150 | 0.7 |
| WY-FS | 0.7 | 0.15 | 7 | 150 | 0.8 |
| WY-HQ | 0.8 | 0.15 | 7 | 150 | 0.8 |
| WY-N | 0.8 | 0.15 | 7 | 150 | 0.8 |
| WY-P | 0.8 | 0.15 | 7 | 150 | 0.8 |

S4 Table 4. Accuracy of the XGBoost Model on the Test Set (2000)

|  | Test set | | | | |
| --- | --- | --- | --- | --- | --- |
| Types | AUC | Weighted F1-Score | Accuracy | Class 0 F1-Score | Class 1 F1-Score |
| CS-HQ | 0.997 | 0.988 | 0.988 | 0.958 | 0.993 |
| CS-N | 0.988 | 0.955 | 0.955 | 0.968 | 0.920 |
| CS-P | 0.989 | 0.956 | 0.956 | 0.970 | 0.922 |
| FS-HQ | 0.997 | 0.984 | 0.984 | 0.990 | 0.958 |
| FS-N | 0.980 | 0.940 | 0.941 | 0.877 | 0.961 |
| FS-P | 0.981 | 0.943 | 0.943 | 0.885 | 0962 |
| N-HQ | 0.951 | 0.892 | 0.894 | 0.932 | 0.758 |
| N-P | 0.934 | 0.943 | 0.944 | 0.572 | 0.970 |
| P-HQ | 0.952 | 0.894 | 0.894 | 0.930 | 0.772 |
| SDR-CS | 0.983 | 0.951 | 0.952 | 0.859 | 0.971 |
| SDR-FS | 0.990 | 0.970 | 0.970 | 0.982 | 0.907 |
| SDR-HQ | 0.994 | 0.978 | 0.978 | 0.928 | 0.987 |
| SDR-N | 0.984 | 0.945 | 0.945 | 0.961 | 0.903 |
| SDR-P | 0.983 | 0.943 | 0.944 | 0.960 | 0.901 |
| SDR-WY | 0.999 | 0.990 | 0.990 | 0.990 | 0.990 |
| WY-CS | 0.988 | 0.948 | 0.948 | 0.950 | 0.946 |
| WY-FS | 0.995 | 0.971 | 0.971 | 0.966 | 0.975 |
| WY-HQ | 0.997 | 0.980 | 0.980 | 0.981 | 0.978 |
| WY-N | 0.982 | 0.944 | 0.944 | 0.904 | 0.960 |
| WY-P | 0.983 | 0.946 | 0.946 | 0.908 | 0.961 |

S4 Table 5. Accuracy of the XGBoost Model on the Test Set (2010)

|  | Test set | | | | |
| --- | --- | --- | --- | --- | --- |
| Types | AUC | Weighted F1-Score | Accuracy | Class 0 F1-Score | Class 1 F1-Score |
| CS-HQ | 0.997 | 0.987 | 0.987 | 0.957 | 0.992 |
| CS-N | 0.990 | 0.962 | 0.962 | 0.973 | 0.934 |
| CS-P | 0.988 | 0.958 | 0.958 | 0.970 | 0.926 |
| FS-HQ | 0.996 | 0.980 | 0.980 | 0.988 | 0.951 |
| FS-N | 0.984 | 0.949 | 0.949 | 0.894 | 0.966 |
| FS-P | 0.985 | 0.950 | 0.951 | 0.900 | 0.967 |
| N-HQ | 0.950 | 0.895 | 0.896 | 0.934 | 0.759 |
| N-P | 0.919 | 0.942 | 0.944 | 0.532 | 0.970 |
| P-HQ | 0.944 | 0.889 | 0.890 | 0.931 | 0.739 |
| SDR-CS | 0.974 | 0.939 | 0.940 | 0.832 | 0.963 |
| SDR-FS | 0.985 | 0.960 | 0.961 | 0.976 | 0.884 |
| SDR-HQ | 0.987 | 0.964 | 0.964 | 0.885 | 0.979 |
| SDR-N | 0.979 | 0.937 | 0.938 | 0.956 | 0.892 |
| SDR-P | 0.977 | 0.934 | 0.934 | 0.954 | 0.885 |
| SDR-WY | 0.998 | 0.984 | 0.984 | 0.982 | 0.985 |
| WY-CS | 0.988 | 0.949 | 0.950 | 0.938 | 0.950 |
| WY-FS | 0.993 | 0.968 | 0.968 | 0.969 | 0.966 |
| WY-HQ | 0.996 | 0.975 | 0.975 | 0.971 | 0.978 |
| WY-N | 0.984 | 0.944 | 0944 | 0.926 | 0.955 |
| WY-P | 0.985 | 0.943 | 0.943 | 0.924 | 0.954 |

S4 Table 6. Accuracy of the XGBoost Model on the Test Set (2020)

|  | Test set | | | | |
| --- | --- | --- | --- | --- | --- |
| Types | AUC | Weighted F1-Score | Accuracy | Class 0 F1-Score | Class 1 F1-Score |
| CS-FS | 0.995 | 0.978 | 0.978 | 0.986 | 0.944 |
| CS-HQ | 0.996 | 0.988 | 0.988 | 0.956 | 0.993 |
| CS-N | 0.992 | 0.964 | 0.964 | 0.975 | 0.935 |
| CS-P | 0.992 | 0.964 | 0.964 | 0.975 | 0.935 |
| FS-HQ | 0.997 | 0.983 | 0.984 | 0.989 | 0.963 |
| FS-N | 0.987 | 0.954 | 0.954 | 0.900 | 0.970 |
| FS-P | 0.988 | 0.955 | 0.955 | 0.904 | 0.970 |
| N-HQ | 0.954 | 0.886 | 0.886 | 0.920 | 0.797 |
| N-P | 0.916 | 0.940 | 0.941 | 0.523 | 0.968 |
| P-HQ | 0.948 | 0.880 | 0.878 | 0.914 | 0.789 |
| SDR--CS | 0.980 | 0.948 | 0.948 | 0.847 | 0.969 |
| SDR-FS | 0.987 | 0.963 | 0.963 | 0.977 | 0.905 |
| SDR-HQ | 0.993 | 0.974 | 0.974 | 0.914 | 0.985 |
| SDR-N | 0.982 | 0.949 | 0.949 | 0.965 | 0.907 |
| SDR-P | 0.983 | 0.946 | 0.946 | 0.963 | 0.903 |
| SDR-WY | 0.998 | 0.984 | 0.984 | 0.983 | 0.984 |
| WY-CS | 0.989 | 0.956 | 0.956 | 0.962 | 0.946 |
| WY-FS | 0.993 | 0.965 | 0.965 | 0.960 | 0.969 |
| WY-HQ | 0.996 | 0.978 | 0.978 | 0.981 | 0.974 |
| WY-N | 0.982 | 0.946 | 0.946 | 0.903 | 0.962 |
| WY-P | 0.982 | 0.941 | 0.941 | 0.894 | 0.959 |
