# Supplementary material for: Identifying driving mechanisms and threshold effects of trade-offs and synergies among ecosystem services: A case study of Henan Province, China
Source: PLoS One. 2026 Apr 21;21(4):e0347200. doi: 10.1371/journal.pone.0347200 (PMC13099101; doi:10.1371/journal.pone.0347200)
Supplement: S2 Table — (DOCX) [file pone.0347200.s009.docx]

S1 Table 2. p-values and FDR-adjusted p-values for each service type

|  | 2000 | | 2010 | | 2020 | |
| --- | --- | --- | --- | --- | --- | --- |
| Types | p-values | FDR-adjusted p-values | p-values | FDR-adjusted p-values | p-values | FDR-adjusted p-values |
| HQ-SDR | 0.000 | 0.000 | 0.000 | 0.000 | 0.000 | 0.000 |
| HQ-CS | 0.000 | 0.000 | 0.000 | 0.000 | 0.000 | 0.000 |
| HQ-FS | <0.001 | 0.00525 | <0.001 | 0.00525 | <0.001 | 0.00525 |
| HQ-N | <0.001 | 0.00525 | <0.001 | 0.00525 | <0.001 | 0.00525 |
| HQ-P | <0.001 | 0.00525 | <0.001 | 0.00525 | <0.001 | 0.00525 |
| HQ-WY | <0.001 | 0.00525 | 0.234 | 0.2457 | <0.001 | 0.00525 |
| SDR-CS | <0.001 | 0.00525 | <0.001 | 0.00525 | <0.001 | 0.00525 |
| SDR-FS | <0.001 | 0.00525 | <0.001 | 0.00525 | <0.001 | 0.00525 |
| SDR-N | <0.001 | 0.00525 | <0.001 | 0.00525 | <0.001 | 0.00525 |
| SDR-P | <0.001 | 0.00525 | <0.001 | 0.00525 | <0.001 | 0.00525 |
| SDR-WY | <0.001 | 0.00525 | <0.001 | 0.00525 | <0.001 | 0.00525 |
| CS-FS | 0.014 | 0.014 | 0.044 | 0.0486 | 0.537 | 0.537 |
| CS-N | <0.001 | 0.00525 | <0.001 | 0.00525 | <0.001 | 0.00525 |
| CS-P | <0.001 | 0.00525 | <0.001 | 0.00525 | <0.001 | 0.00525 |
| CS-WY | <0.001 | 0.00525 | <0.001 | 0.00525 | <0.001 | 0.00525 |
| FS-N | <0.001 | 0.00525 | <0.001 | 0.00525 | <0.001 | 0.00525 |
| FS-P | <0.001 | 0.00525 | <0.001 | 0.00525 | <0.001 | 0.00525 |
| FS-WY | <0.001 | 0.00525 | 0.426 | 0.426 | 0.010 | 0.0105 |
| N-P | 0.000 | 0.000 | 0.000 | 0.000 | 0.000 | 0.000 |
| N-WY | <0.001 | 0.00525 | <0.001 | 0.00525 | <0.001 | 0.00525 |
| P-WY | <0.001 | 0.00525 | <0.001 | 0.00525 | <0.001 | 0.00525 |
