# Supplementary material for: Identifying driving mechanisms and threshold effects of trade-offs and synergies among ecosystem services: A case study of Henan Province, China
Source: PLoS One. 2026 Apr 21;21(4):e0347200. doi: 10.1371/journal.pone.0347200 (PMC13099101; doi:10.1371/journal.pone.0347200)
Supplement: S3 Table — (DOCX) [file pone.0347200.s010.docx]

S2 Table 1. State classification and range division of each node in the BBN-ESs Model in 2000

| Nodes | State and scope（2000） | | | | Unit |
| --- | --- | --- | --- | --- | --- |
|  | low | medium | high | highest |  |
| Population | [0,606) | [606,3522) | [3522,12152) | [12152,47777] | People/km^2^ |
| Slope | [0,1.4) | [1.44,4.2) | [4.2,8.5) | [8.5,24.4] | ° |
| Precipitation | [549.6,708.4) | [708.4,860.5) | [860.9,1027.8) | [1027.8,1478.3] | mm |
| Land Use | cropland, forestland, grassland, waters, building, others | | | | — |
| Temperature | [5.4,11.8) | [11.8,13.8) | [13.8,15.1) | [15.1,16.6] | ℃ |
| Rainfall erosion | [1913.5,2965.1) | [2965.1,4061.7) | [4061.7,5388.8) | [5388.8,9665.6] | MJ·mm/(ha·h·a) |
| Soil erosion | [0.0.010) | [0.010,0.014) | [0.014,0.018) | [0.018,0.021] | t·ha·h/(ha·MJ·mm) |
| AET | [720.2,1030.6) | [1030.6,1138.4) | [1038.4,1200.4) | [1200.4,1269.0] | mm |
| NDVI | [0,0.5) | [0.5,0.6) | [0.6,0.7) | [0.7,1] | — |
| P | [0,30.3) | [30.3,75.2) | [75.2,120.1) | [120.1,287.4] | kg |
| N | [0,192.6) | [192.6,467.7) | [467.7,742.9) | [724.9,1761.1] | kg |
| HQ | [0,0.4) | [0.4,0.6) | [0.6,0.8) | [0.8,1] | — |
| SDR | [0,27.0) | [27.0,102.6) | [102.6,275.5) | [275.5,1382.8] | kt |
| CS | [0,3640) | [3640,6776) | [6776,8404) | [8404,13353] | t |
| WY | [0,295.4) | [295.4,437.1) | [437.1,598.9) | [598.9,1036.1] | mm |
| FS | [0,151.7) | [151.7,338.1) | [338.1,387.1) | [387.1,525.7] | t |
