# Supplementary material for: Identifying driving mechanisms and threshold effects of trade-offs and synergies among ecosystem services: A case study of Henan Province, China
Source: PLoS One. 2026 Apr 21;21(4):e0347200. doi: 10.1371/journal.pone.0347200 (PMC13099101; doi:10.1371/journal.pone.0347200)
Supplement: S4 Table — (DOCX) [file pone.0347200.s011.docx]

S2 Table 2. State classification and range division of each node in the BBN-ESs Model in 2010

| Nodes | State and scope（2010） | | | | Unit |
| --- | --- | --- | --- | --- | --- |
|  | low | medium | high | highest |  |
| Population | [0,1393) | [1393,5471) | [5472,16703) | [16703,65451] | People/km^2^ |
| Slope | [0,1.4) | [1.44,4.2) | [4.2,8.5) | [8.5,24.4] | ° |
| Precipitation | [547.6,639.3) | [639.3,749.7) | [749.7,900.7) | [900.7,1350.1] | mm |
| Land Use | cropland, forestland, grassland, waters, building, others | | | | — |
| Temperature | [5.6,12.1) | [12.1,14.1) | [14.1,15.3) | [15.3,16.8] | ℃ |
| Rainfall erosion | [1901.7,2488.4) | [2488.4,3236.2) | [3236.2,4356.7) | [4356.7,8253.3] | MJ·mm/(ha·h·a) |
| Soil erosion | [0.0.010) | [0.010,0.014) | [0.014,0.018) | [0.018,0.021] | t·ha·h/(ha·MJ·mm) |
| AET | [722.7,1022.5) | [1022.5,1127.1) | [1127.1,1186.3) | [1186.3,1267.2] | mm |
| NDVI | [0,0.5) | [0.5,0.7) | [0.7,0.8) | [0.8,1] | — |
| P | [0,32.1) | [32.1,77.4) | [77.4,122.8) | [122.8,283.4] | kg |
| N | [0,196.7) | [196.7,474.8) | [474.8,753.1) | [753.1,1736.7] | kg |
| HQ | [0,0.4) | [0.4,0.7) | [0.7,0.8) | [0.9,1] | — |
| SDR | [0,17.9) | [17.9,71.6) | [71.6,175.4) | [175.4,916.3] | kt |
| CS | [0,3640) | [3640,6776) | [6776,8404) | [8404,13353] | t |
| WY | [0,243.1) | [243.1,346.8) | [346.8,489.8) | [489.8,915.4] | mm |
| FS | [0,206.9) | [206.9,455.7) | [455.7,516.9) | [516.9,646.5] | t |
