# Supplementary material for: Identifying driving mechanisms and threshold effects of trade-offs and synergies among ecosystem services: A case study of Henan Province, China
Source: PLoS One. 2026 Apr 21;21(4):e0347200. doi: 10.1371/journal.pone.0347200 (PMC13099101; doi:10.1371/journal.pone.0347200)
Supplement: S5 Table — (DOCX) [file pone.0347200.s012.docx]

S2 Table 3. State classification and range division of each node in the BBN-ESs Model in 2020

| Nodes | State and scope（2020） | | | | Unit |
| --- | --- | --- | --- | --- | --- |
|  | low | medium | high | highest |  |
| Population | [0,2183) | [2183,8694) | [8694,25372) | [25372,78458] | People/km^2^ |
| Slope | [0,1.4) | [1.44,4.2) | [4.2,8.5) | [8.5,24.4] | ° |
| Precipitation | [590.7,719.4) | [719.4,863.5) | [863.5,1043.5) | [1043.5,1552.5] | mm |
| Land Use | cropland, forestland, grassland, waters, building, others | | | | — |
| Temperature | [5.7,12.4) | [12.4,14.5) | [14.5,15.8) | [15.8,17.1] | ℃ |
| Rainfall Erosion | [2151.3,3042.6) | [3042.6,4111.3) | [4111.3,5578.6) | [5578.6,10359.4] | MJ·mm/(ha·h·a) |
| Soil Erosion | [0.0.010) | [0.010,0.014) | [0.014,0.018) | [0.018,0.021] | t·ha·h/(ha·MJ·mm) |
| AET | [726.1,1031.3) | [1031.3,1139.1) | [1139.1,1214.3) | [1214.3,1290.2] | mm |
| NDVI | [0,0.5) | [0.5,0.7) | [0.7,0.8) | [0.8,1] | — |
| P | [0,30.1) | [30.1,75.2) | [75.2,124.2) | [124.2,321.3] | kg |
| N | [0,184.5) | [184.5,461.4) | [461.4,769.1) | [769.1,1968.8] | kg |
| HQ | [0,0.4) | [0.4,0.6) | [0.6,0.8) | [0.8,1] | — |
| SDR | [0,25.4) | [25.4,111.6) | [111.6,334.9) | [334.9,1299.0] | kt |
| CS | [0,3640) | [3640,6776) | [6776,8404) | [8404,13353] | t |
| WY | [0,166.3) | [166.3,297.1) | [297.1,467.4) | [467.4,1014.1] | mm |
| FS | [0,266.8) | [266.8,587.7) | [587.7,673.3) | [673.3,847.9] | t |
