# Supplementary material for: Identifying driving mechanisms and threshold effects of trade-offs and synergies among ecosystem services: A case study of Henan Province, China
Source: PLoS One. 2026 Apr 21;21(4):e0347200. doi: 10.1371/journal.pone.0347200 (PMC13099101; doi:10.1371/journal.pone.0347200)
Supplement: S6 Table — (DOCX) [file pone.0347200.s013.docx]

S4 Table 1. Optimized Parameters for the XGBoost Model (2000)

| Parameter Types | | | | | |
| --- | --- | --- | --- | --- | --- |
| Types | colsample_bytree | learning_rate | max_depth | n_estimators | subsample |
| CS-HQ | 0.8 | 0.15 | 7 | 150 | 0.8 |
| CS-N | 0.7 | 0.15 | 7 | 150 | 0.8 |
| CS-P | 0.7 | 0.15 | 7 | 150 | 0.7 |
| FS-HQ | 0.8 | 0.1 | 7 | 150 | 0.8 |
| FS-N | 0.8 | 0.15 | 7 | 150 | 0.7 |
| FS-P | 0.8 | 0.15 | 7 | 150 | 0.8 |
| N-HQ | 0.8 | 0.15 | 7 | 150 | 0.8 |
| N-P | 0.8 | 0.15 | 7 | 200 | 0.8 |
| P-HQ | 0.7 | 0.15 | 7 | 150 | 0.8 |
| SDR-CS | 0.8 | 0.15 | 7 | 150 | 0.8 |
| SDR-FS | 0.8 | 0.15 | 7 | 150 | 0.8 |
| SDR-HQ | 0.7 | 0.15 | 7 | 150 | 0.8 |
| SDR-N | 0.8 | 0.15 | 7 | 150 | 0.8 |
| SDR-P | 0.8 | 0.15 | 7 | 150 | 0.8 |
| SDR-WY | 0.8 | 0.4 | 6 | 150 | 0.8 |
| WY-CS | 0.8 | 0.15 | 7 | 150 | 0.7 |
| WY-FS | 0.8 | 0.15 | 7 | 150 | 0.8 |
| WY-HQ | 0.8 | 0.15 | 7 | 150 | 0.7 |
| WY-N | 0.8 | 0.15 | 7 | 150 | 0.8 |
| WY-P | 0.7 | 0.15 | 7 | 150 | 0.8 |
