# Supplementary material for: Identifying driving mechanisms and threshold effects of trade-offs and synergies among ecosystem services: A case study of Henan Province, China
Source: PLoS One. 2026 Apr 21;21(4):e0347200. doi: 10.1371/journal.pone.0347200 (PMC13099101; doi:10.1371/journal.pone.0347200)
Supplement: S9 Table — (DOCX) [file pone.0347200.s016.docx]

S4 Table 4. Accuracy of the XGBoost Model on the Test Set (2000)

|  | Test set | | | | |
| --- | --- | --- | --- | --- | --- |
| Types | AUC | Weighted F1-Score | Accuracy | Class 0 F1-Score | Class 1 F1-Score |
| CS-HQ | 0.997 | 0.988 | 0.988 | 0.958 | 0.993 |
| CS-N | 0.988 | 0.955 | 0.955 | 0.968 | 0.920 |
| CS-P | 0.989 | 0.956 | 0.956 | 0.970 | 0.922 |
| FS-HQ | 0.997 | 0.984 | 0.984 | 0.990 | 0.958 |
| FS-N | 0.980 | 0.940 | 0.941 | 0.877 | 0.961 |
| FS-P | 0.981 | 0.943 | 0.943 | 0.885 | 0962 |
| N-HQ | 0.951 | 0.892 | 0.894 | 0.932 | 0.758 |
| N-P | 0.934 | 0.943 | 0.944 | 0.572 | 0.970 |
| P-HQ | 0.952 | 0.894 | 0.894 | 0.930 | 0.772 |
| SDR-CS | 0.983 | 0.951 | 0.952 | 0.859 | 0.971 |
| SDR-FS | 0.990 | 0.970 | 0.970 | 0.982 | 0.907 |
| SDR-HQ | 0.994 | 0.978 | 0.978 | 0.928 | 0.987 |
| SDR-N | 0.984 | 0.945 | 0.945 | 0.961 | 0.903 |
| SDR-P | 0.983 | 0.943 | 0.944 | 0.960 | 0.901 |
| SDR-WY | 0.999 | 0.990 | 0.990 | 0.990 | 0.990 |
| WY-CS | 0.988 | 0.948 | 0.948 | 0.950 | 0.946 |
| WY-FS | 0.995 | 0.971 | 0.971 | 0.966 | 0.975 |
| WY-HQ | 0.997 | 0.980 | 0.980 | 0.981 | 0.978 |
| WY-N | 0.982 | 0.944 | 0.944 | 0.904 | 0.960 |
| WY-P | 0.983 | 0.946 | 0.946 | 0.908 | 0.961 |
