# Supplementary material for: Identifying driving mechanisms and threshold effects of trade-offs and synergies among ecosystem services: A case study of Henan Province, China
Source: PLoS One. 2026 Apr 21;21(4):e0347200. doi: 10.1371/journal.pone.0347200 (PMC13099101; doi:10.1371/journal.pone.0347200)
Supplement: S10 Table — (DOCX) [file pone.0347200.s017.docx]

S4 Table 5. Accuracy of the XGBoost Model on the Test Set (2010)

|  | Test set | | | | |
| --- | --- | --- | --- | --- | --- |
| Types | AUC | Weighted F1-Score | Accuracy | Class 0 F1-Score | Class 1 F1-Score |
| CS-HQ | 0.997 | 0.987 | 0.987 | 0.957 | 0.992 |
| CS-N | 0.990 | 0.962 | 0.962 | 0.973 | 0.934 |
| CS-P | 0.988 | 0.958 | 0.958 | 0.970 | 0.926 |
| FS-HQ | 0.996 | 0.980 | 0.980 | 0.988 | 0.951 |
| FS-N | 0.984 | 0.949 | 0.949 | 0.894 | 0.966 |
| FS-P | 0.985 | 0.950 | 0.951 | 0.900 | 0.967 |
| N-HQ | 0.950 | 0.895 | 0.896 | 0.934 | 0.759 |
| N-P | 0.919 | 0.942 | 0.944 | 0.532 | 0.970 |
| P-HQ | 0.944 | 0.889 | 0.890 | 0.931 | 0.739 |
| SDR-CS | 0.974 | 0.939 | 0.940 | 0.832 | 0.963 |
| SDR-FS | 0.985 | 0.960 | 0.961 | 0.976 | 0.884 |
| SDR-HQ | 0.987 | 0.964 | 0.964 | 0.885 | 0.979 |
| SDR-N | 0.979 | 0.937 | 0.938 | 0.956 | 0.892 |
| SDR-P | 0.977 | 0.934 | 0.934 | 0.954 | 0.885 |
| SDR-WY | 0.998 | 0.984 | 0.984 | 0.982 | 0.985 |
| WY-CS | 0.988 | 0.949 | 0.950 | 0.938 | 0.950 |
| WY-FS | 0.993 | 0.968 | 0.968 | 0.969 | 0.966 |
| WY-HQ | 0.996 | 0.975 | 0.975 | 0.971 | 0.978 |
| WY-N | 0.984 | 0.944 | 0944 | 0.926 | 0.955 |
| WY-P | 0.985 | 0.943 | 0.943 | 0.924 | 0.954 |
