# Supplementary material for: Identifying driving mechanisms and threshold effects of trade-offs and synergies among ecosystem services: A case study of Henan Province, China
Source: PLoS One. 2026 Apr 21;21(4):e0347200. doi: 10.1371/journal.pone.0347200 (PMC13099101; doi:10.1371/journal.pone.0347200)
Supplement: S11 Table — (DOCX) [file pone.0347200.s018.docx]

S4 Table 6. Accuracy of the XGBoost Model on the Test Set (2020)

|  | Test set | | | | |
| --- | --- | --- | --- | --- | --- |
| Types | AUC | Weighted F1-Score | Accuracy | Class 0 F1-Score | Class 1 F1-Score |
| CS-FS | 0.995 | 0.978 | 0.978 | 0.986 | 0.944 |
| CS-HQ | 0.996 | 0.988 | 0.988 | 0.956 | 0.993 |
| CS-N | 0.992 | 0.964 | 0.964 | 0.975 | 0.935 |
| CS-P | 0.992 | 0.964 | 0.964 | 0.975 | 0.935 |
| FS-HQ | 0.997 | 0.983 | 0.984 | 0.989 | 0.963 |
| FS-N | 0.987 | 0.954 | 0.954 | 0.900 | 0.970 |
| FS-P | 0.988 | 0.955 | 0.955 | 0.904 | 0.970 |
| N-HQ | 0.954 | 0.886 | 0.886 | 0.920 | 0.797 |
| N-P | 0.916 | 0.940 | 0.941 | 0.523 | 0.968 |
| P-HQ | 0.948 | 0.880 | 0.878 | 0.914 | 0.789 |
| SDR--CS | 0.980 | 0.948 | 0.948 | 0.847 | 0.969 |
| SDR-FS | 0.987 | 0.963 | 0.963 | 0.977 | 0.905 |
| SDR-HQ | 0.993 | 0.974 | 0.974 | 0.914 | 0.985 |
| SDR-N | 0.982 | 0.949 | 0.949 | 0.965 | 0.907 |
| SDR-P | 0.983 | 0.946 | 0.946 | 0.963 | 0.903 |
| SDR-WY | 0.998 | 0.984 | 0.984 | 0.983 | 0.984 |
| WY-CS | 0.989 | 0.956 | 0.956 | 0.962 | 0.946 |
| WY-FS | 0.993 | 0.965 | 0.965 | 0.960 | 0.969 |
| WY-HQ | 0.996 | 0.978 | 0.978 | 0.981 | 0.974 |
| WY-N | 0.982 | 0.946 | 0.946 | 0.903 | 0.962 |
| WY-P | 0.982 | 0.941 | 0.941 | 0.894 | 0.959 |
